# Swarming caddisflies in the mid-cretaceous

**DOI:** 10.1093/nsr/nwae227

**Published:** 2024-06-28

**Authors:** Jiajia Wang, Michael S Engel, Weiting Zhang, Chungkun Shih, Rui Qiu, Dong Ren

**Affiliations:** College of Life Sciences, Capital Normal University, Beijing 100048, China; Key Laboratory of Zoological Systematics and Evolution, Institute of Zoology, Chinese Academy of Sciences, Beijing 100101, China; Division of Invertebrate Zoology, American Museum of Natural History, New York 10024, USA; Facultad de Ciencias Biológicas, Universidad Nacional Mayor de San Marcos, Lima 7345/7445, Perú; Departamento de Entomología, Museo de Historia Natural, Universidad Nacional Mayor de San Marcos, Lima 7345/7445, Perú; Hebei International Joint Research Center for Paleoanthropology, Hebei GEO University, Shijiazhuang 050031, China; College of Life Sciences, Capital Normal University, Beijing 100048, China; Department of Paleobiology, National Museum of Natural History, Smithsonian Institution, Washington, DC 20013, USA; National Natural History Museum of China, Beijing 100050, China; College of Life Sciences, Capital Normal University, Beijing 100048, China

**Keywords:** swarming, caddisflies, Cretaceous, ancestral-trait reconstructions

## Abstract

Swarming, as a special form of mating aggregation, is most noteworthy in insects of the orders Ephemeroptera, Diptera, and Trichoptera. Swarming in extant trichopterans is well understood in terms of sex composition, specific mating behaviors, and functional morphological specializations of adults, but an exploration of the evolution of such aggregative behaviors is hampered by the dearth of available examples from the fossil record as well as the ability to reliably distinguish the few gatherings as the result of swarming relative to other taphonomic or behavioral factors. Herein we describe five new fossil species of caddisflies preserved in mid-Cretaceous amber from Myanmar, all preserved as large aggregations. Monospecific aggregations of these five new species can be positively identified as swarms based on morphological traits of wing shape, as well as the presence of particular forms of sexual dimorphism. Results of a phylogenetic reconstruction of both molecular and morphological data as well as ancestral-trait reconstructions and tip-dating analyses indicate that swarming was likely present in the Triassic as a feature of the trichopteran groundplan. Since most Mesozoic insectivorous predators were diurnal based on morphological evidence, largely nocturnal caddisflies would have been freed from such pressures. The phylogeny also shows a correlation between the rise of nocturnal bat predators from the Paleocene or early Eocene and the repeated loss of swarming from various clades of caddisflies, revealing the potential impact of bat predation on reshaping the behavioral landscape of Trichoptera during the Cenozoic.

## Teaser

The amber fossils show that the swarming behavior of Trichoptera already existed during the Cretaceous.

## INTRODUCTION

The gathering of insects is a common phenomenon for the purposes of feeding, positive phototaxis, mass migration, clustering in winter, simultaneous emergence, or swarms for mating [1–5]. Determining the reasons for aggregating in fossil material, however, can at times be challenging if not impossible, and may often resort to nothing more than idle speculation. Preservation in amber allows fine details of small animals to be observed with high fidelity, and also permits a more robust exploration of behaviors from life, ranging from feeding, gathering, mating, mutualisms, commensalisms, or parasitism [6–8]. Three fossil records of gathering have been identified or mentioned as the result of swarming: whiteflies in Eocene Baltic amber [9], caddisflies of *Palerasnitsynus* in mid-Cretaceous Kachin amber [10], and mayflies of the Sharephemeridae (Ephemeroptera) in the Jurassic Shiti Formation of South China [11]. Little evidence was available in each case as to why the insects were interpreted as gathering for swarms, versus other forms of aggregation. In order to identify this special behavior from fossils here we report several exceptional cases from six pieces of mid-Cretaceous Kachin amber pieces with significant aggregations of caddisflies, all of which preserved a considerable suite of evidence for swarming.

## RESULTS AND DISCUSSION

### Comparisons and morphological evidence

**Figure 1.**
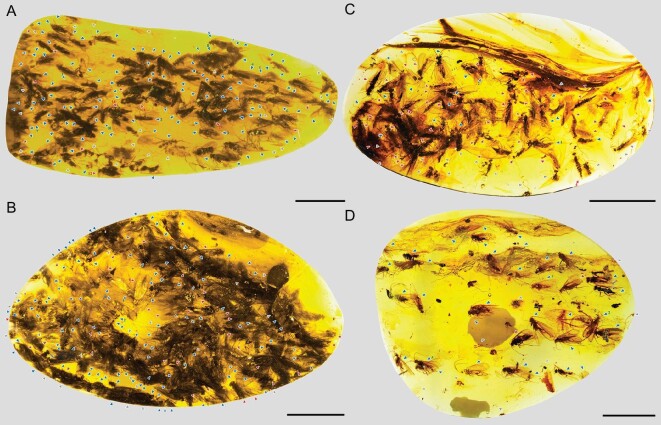
Swarming caddisflies. (A) *Copulariella ramus* gen. et sp. nov., holotype, CNU-TRI-MA-2015503, males and females. (B) *Palerasnitsynus queqiaoi* sp. nov., holotype, CNU-TRI-MA-2015504, males and females. (C) *Palerasnitsynus qixi* sp. nov., holotype, CNU-TRI-MA-2015505, males and females. (D) *Palerasnitsynus aggregatus* sp. nov., holotype, CNU-TRI-MA-2015506, males and females. Males are indicated by blue arrows, females with red arrows, and sex-indeterminate individuals with grey arrows. Numbers represent individuals measured in Tables S2–S3. Scale bars represent 5000 μm in (A–D).

The obvious aggregations, a large gathering of adult caddisflies, were found in six small amber pieces (CNU-TRI-MA-2015503 to CNU-TRI-MA-2015508). A total of 187 individuals of *Copulariella ramus*, including 139 males, 20 females, and 28 sex-indeterminate individuals, are preserved in a relatively small piece of amber of ∼34 × 16 × 3 mm (CNU-TRI-MA-2015503, Fig. 1A, Figs S1 and S2A). A total of 243 individuals of *Palerasnitsynus queqiaoi*, including 161 males, 54 females, and 28 sex-indeterminate individuals, are preserved in a relatively large piece of amber of ∼30 × 17 × 3.5 mm (CNU-TRI-MA-2015504, Fig. 1B and Fig. S2B). A total of 117 individuals of *P. qixi*, including 23 males, 68 females, and 26 sex-indeterminate individuals, are preserved in a piece of amber of ∼24 × 14 × 3 mm (CNU-TRI-MA-2015505, Fig. 1C and Fig. S2C). A total of 43 individuals of *P. aggregatus*, including 33 males, two females, and eight sex-indeterminate individuals, are preserved in a piece of amber of ∼25 × 20 × 5 mm (CNU-TRI-MA-2015506, Fig. 1D and Fig. S2D). Another aggregation of 157 individuals of *P. xiuqiu*, including 143 males and 14 sex-indeterminate individuals, are preserved in a piece of amber of ∼24 × 16 × 2.5 mm (CNU-TRI-MA-2015507, Fig. S3A and B). A total of 27 males of *P. aggregatus* are preserved in a relatively small piece of amber of ∼31 × 17 × 2 mm (CNU-TRI-MA-2015508, Fig. S3C and D).

**Figure 2.**
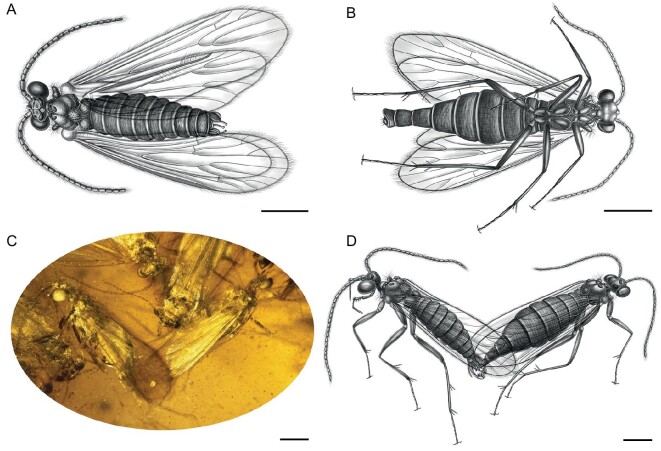
(A) Line drawing of male *Copulariella ramus*. (B) Line drawing of female *C. ramus*. (C) Mating pair of *Palerasnitsynus queqiao*, male (left) and female (right). (D) Line drawing of male (left) and female (right) of mating *P. queqiaoi*. Scale bars represent 500 μm in (A–D).

The aggregations cannot be the result of feeding swarms as the mouthparts of all individuals of all species appear to be vestigial (Figs 1, 2, and Fig. S2), lacking the ability to feed, as in most adult trichopterans [12,13]. Similarly, these are not overwintering associations owing to the perpetual tropical conditions of the environment under which the Myanmar resins were exuded during the mid-Cretaceous [14]. Mass emergences can also be ruled out as a source for the aggregations owing to the different abrasion patterns of setae on the wings. The individuals in the pieces possess differently colored wings—ranging from dark gray to nearly transparent and variations in-between—resulting from different degrees of setal abrasion. In extant Trichoptera, these color differences are largely the result of abrasion of the setae or scales [15,16], with the variation in wing wear representing different ages of individuals. The fossil aggregations are consistent with associations of individuals of varied ages and wear and their grouping together is therefore not the result of simultaneous emergence, which does occur in some extant insect species [17].

The male sex ratio (males/(males + females)) in the aggregations of *C. ramus, P. queqiaoi, P. qixi, P. aggregatus*, and *P. xiuqiu* are 0.87, 0.75, 0.25, 0.95 (1.00 in Fig S3C, D), and 1.00, respectively. With the exception of the last piece, all of the fossil aggregations preserve ratios that significantly deviate from 1, a pattern shared with most extant swarming insects whose sex ratios nearly always exceed 0.6 [4,5,18,19].

Some characters on the genitalia of these caddisflies indicate they were in a breeding period. All of the inferior appendages of the males are elongate, and the same has been observed in other swarming caddisfly specimens preserved in Cretaceous amber [10]. When compared to the inferior appendages of extant trichopterans, species with known swarming behavior always have males with a relatively elongate inferior appendage or their genitalia are beset with more complex spines. The more elongate arcuate inferior appendages allow males to more assuredly attach to the females during mating, increasing the probability of successful copulation [20]. All the phalli of the preserved males are extended and clearly visible, longer than the preanal appendages in length (Figs 1, 2 and Figs S2, S3). In particular, we found that the phalli of *P. queqiao* were similarly exposed and males and females were in mating positions (Fig. 2C, D). The abdomens of all females seem to be swollen, and wider than those of the corresponding males (Figs 1, 2 and Figs S2–S4). The abdominal shape of females is consistent with the second developmental stage (of four) for females of extant caddisflies, representing stages from emergence from the cocoon to oviposition during reproductive swarming [21], and indicates that these females would mate soon. The male and female in the specimen are in different preservation states (Fig. 1), and some are in a takeoff position, indicating a high possibility of leaving the group and completing mating near water.

**Figure 3.**
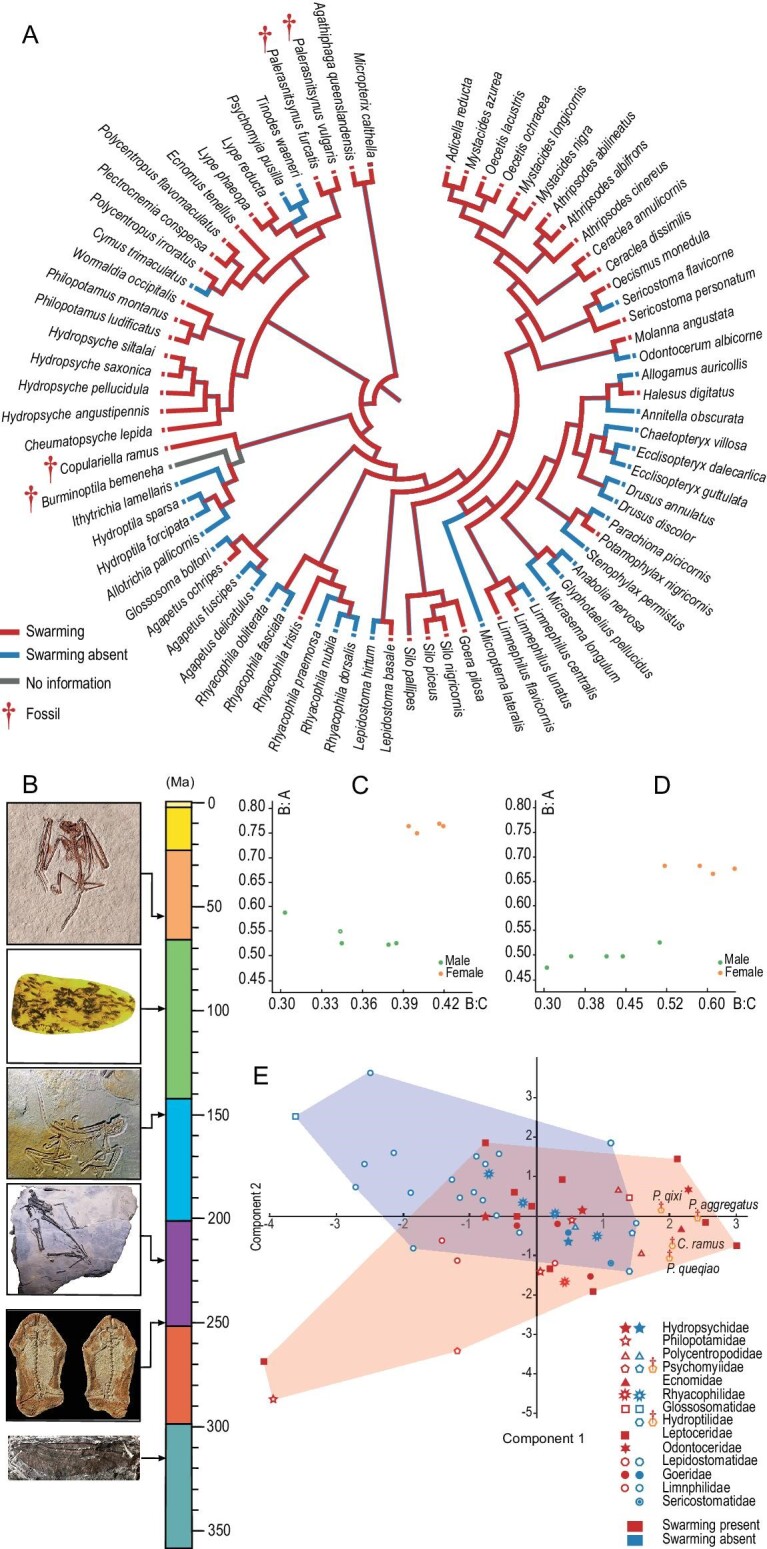
(A) Swarming species. (B) The earliest fossil records of bat (*Icaronycteris* from Rietbergen *et al*. [67]), swarming caddisfly (*Copulariella*), bird (*Archaeopteryx* from Rauhut *et al*. [68]), pterosaur (*Caviramus* from Stecher [69]), frog (*Triadobatrachus* from Ascarrunz *et al*. [70]), and dragonfly (*Erasipteron* from Yang *et al*. [71]). (C) Scatter diagram of compound eyes of *Copulariella ramus*. A, length of eye; B, width of eye; C, distance between eyes. B:A is related to the shape of the compound eyes; B:C is related to the size of compound eyes relative to the size of the head. (D) The scatter diagram of the compound eyes of *Palerasnitsynus queqiaoi*. Data are documented in Dataset S1. (E) Principal component analysis (PCA) of extant and Cretaceous swarming caddisflies based on morphological characters. Red marks represent swarming groups, blue marks represent those where swarming is absent. (PCA analysis explains 62% of the variance by the first principal component and 27% by the second principal component). The chosen species are from nearly all superfamilies of Trichoptera. Data are documented in Tables S4–S6.

The preserved males and females exhibit sexual dimorphism, reflecting their mating and reproductive behaviors [22], and may make it easier, along with chemical cues, for males to locate and identify females within the cloud of individuals of the aggregation, just as in extant swarming species. In all amber pieces with both sexes preserved, the males have relatively longer and wider compound eyes extending beyond or flush with the posterior edge of the head and with a lateral inclination caudally, making them easily distinguishable from females (Fig. 3C, D), and also reflective of increased visual acuity for locating females during swarming, just as in their modern counterparts, such as Hydropsychidae and Leptoceridae [3,22]. In species whose aggregations are dominated by males the antennae are sexually dimorphic (Fig. 3). Antennae of males are relatively stouter, which might provide greater surface areas for olfactory sensilla for finer sensitivity to chemical cues exuded by females, and to putatively improve their detection of females during swarming, as in some extant species [22]. It is interesting that sexually dimorphic antennae are not obvious in *P. qixi* (Fig. 1C and Fig. S2C), the sole species in which the aggregation is dominated by females. The wings of the new species are also sexually dimorphic. In order to accurately describe these differences, we conducted a principal component analysis (PCA) largely based on morphological data from the wings of males and females of the new species. The results indicate that all of the new species are clustered with other taxa exhibiting swarming behavior and they share a similar wing shape (red symbols in Fig. 3E). The male wings of the five new species (Figs 1, 2 and Figs S2, S3) and other swarming species possess a relatively wide wing with rounded apices, while the wings of the males in species lacking swarming behavior are relatively narrow and sharp (blue symbols in Fig. 3E).

It should be noted that in all of the new species, the ratio between the wing length and the body length in males is larger than that of females, demonstrating that the wings of males are relatively longer than those of females. This condition is unusual among caddisflies in that most species possess a longer wing in females since they carry the weight of the eggs [22]. Nonetheless, the condition observed in the fossils is seen in species with a fierce competition among males for mating, with males requiring elongate wings for higher speed and agility in locating and competing for a mate [2,3,16]. The elongate wings of the Cretaceous caddisfly species suggest that mate competition may also have been quite fierce and is further evidence that the aggregations are the result of mating swarms.

Given these above, all of the fossil species are most likely congregated within their individual amber pieces as a result of swarms coming into contact with exuded resin. Collectively, all of the data presented here indicate that these fossil species represent the earliest record of swarming for Trichoptera, demonstrating that this behavior was present at least by the mid-Cretaceous, and these behaviors may have already been present in the early evolution of the order.

Adult caddisflies with small body sizes from Kachin amber are striking and have been described as extinct ‘microcaddisflies’ [10]. All of the caddisflies preserved in Kachin amber reported here show that the body lengths of male individuals are shorter than those of females, even though the wing lengths are approximately subequal for both sexes (Table S3). In addition, males’ compound eyes are statistically larger than females’ (Fig. 3 and Table S2). The combination of characteristics suggests that these males might have flown in vertical zigzag patterns to find females resting on nearby leaves during swarming, as in extant species. This is also partially supported by the preservation of leaves in CNU-TRI-MA-2015505.

### Ancestral-trait reconstruction of swarming behavior

**Figure 4.**
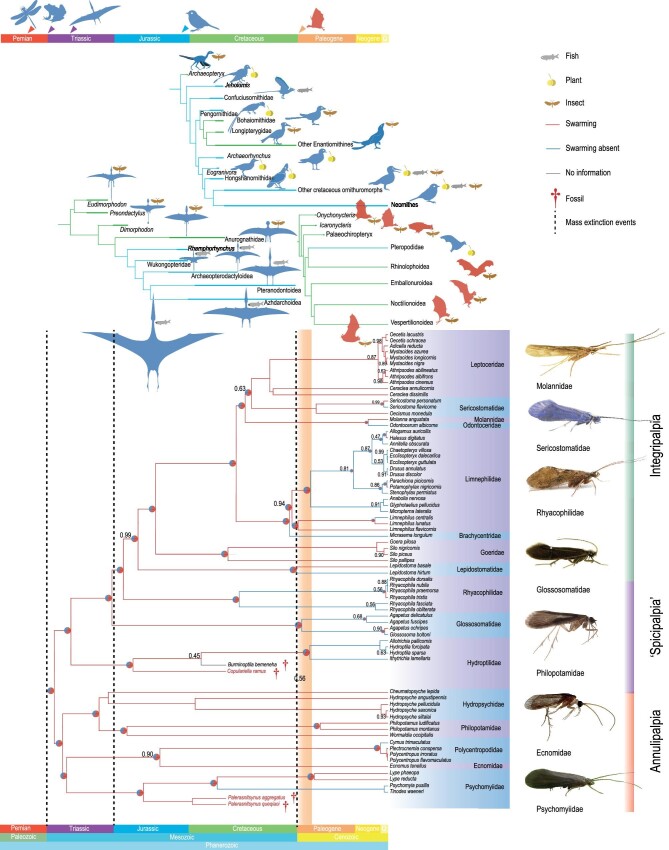
Phylogenetic analysis and ancestral reconstruction of swarming behavior for Trichoptera and their main predators. The ancestral reconstruction of swarming behavior based on morphological and molecular data using a Bayesian total-evidenced dating method with a molecular backbone constraint. The node support (the number at each node) is the posterior probability (only below 1.00). The data source of trichopteran species comes from GenBank and NCBI. The feeding habits sources and phylogeny of pterosaurs, birds, and bats are based on Ősi [30], Bestwick *et al*. [34], O'Connor *et al*. [38], Sigé *et al*. [41], and Storch *et al*. [44]. All figure sources of extant trichopterans are derived from eakringbirds.com and Creative Commons Attribution 4.0 International License. Data sources: Supplementary Data 3 and Supplementary material.

Most extant psychomyiid and hydroptilid species do not swarm. In addition, an absence of swarming behavior also occurs in some extant species of Rhyacophilidae, Hydroptilidae, Glossosomatidae, and Limnephilidae [23], most of which are regarded as basal groups of Integripalpia or belonging to the ‘Spicipalpia’ [24]. From the seeming phylogenetic occurrence of these behaviors, the gain and loss of swarming in Trichoptera would appear to be complex. In order to reconstruct patterns of swarming during trichopteran evolution, a Bayesian Evolutionary Analysis Sampling Tree was undertaken based on morphological and molecular data (Fig. 4). We selected 79 trichopteran species from 15 families, which include the major superfamilies and swarming groups. The phylogeny was estimated from 30 morphological characters and six molecular sequences, specifically 28S, 18S, CAD, EF1a, IDH, and COI (Supplementary Information).

As demonstrated by recent phylogenetic analyses for Trichoptera [24–26], ‘spicipalpians’ are a grade at the base of Integripalpia, rather than an early-diverging lineage of Trichoptera [27]. The dates of occurrence of most trichopteran lineages are similar to those estimated by the node dating [24,27]. Most annulipalpian families diverged during the Triassic and Jurassic, while most integripalpian families originated in the Cretaceous. Our phylogenetic results (Fig. 4) found that *C. ramus, P. queqiaoi, P. qixi, P. aggregatus*, and *P. xiuqiu* are basal among Hydroptilidae and Psychomyiidae, respectively. *Copulariella ramus* belongs to Hydroptilidae, which is supported by the following synapomorphies: apical fork V absent, pentamerous maxillary palpi without annulation, and tibial spur formula 0/2/4 or 0/2/3. *Copulariella ramus* was distinguished from extant hydroptilids by the presence of ocelli, tibial spur formula 0/2/4, a pair of oval setal warts on the mesoscutum, and a rounded tip for both the forewing and hind wing, traits shared symplesiomorphically with other basal integripalpian families (‘Spicipalpia’). *Palerasnitsynus queqiaoi, P. qixi, P. aggregatus*, and *P. xiuqiu* belong to Psychomyiidae as evidenced by the synapomorphies of adults without ocelli, an antenna as long as the forewing, pentamerous and annulated maxillary palpi, maxillary palpomere II subequal to palpomere I in length, tibial spur formula 2/4/4, fork I absent in forewing and hind wing, and mesoscutum covered with two ovoid setal warts. These species are excluded from extant psychomyiids by the mesoscutellum possessing a large and round setal wart that is shared with Polycentropodidae, rather than two semicircular setal warts found in crown-Psychomyiidae, the apex of maxillary palpomere III bearing dark and apicolateral spines and fork III absent in forewing and hind wing.

Swarming behavior was reconstructed as a synapomorphy of Trichoptera based on the ancestral-trait reconstruction. Among Annulipalpia, this behavior is only absent in some psychomyiids and a few polycentropodids, and obviously plesiomorphic for the annulipalpian group as a whole. Swarming behavior gradually disappeared in some lineages of Integripalpia, especially in Rhyacophilidae, Hydroptilidae, Glossosomatidae, Lepidostomatidae, Limnephilidae, and Odontoceridae. Though some species of these families possess swarming behavior, the ancestral-trait reconstruction indicates that the presence of this behavior emerged secondarily in each of these groups and ancestral trichopterans in the Triassic might have possessed swarming behavior (Fig. 4).

### Relationships and paleobiology of swarming behavior

The earlier origin of swarming behavior in caddisflies might have been a specialization for the climate of the Triassic. Synchronous aggregating of sexes would have been advantageous since these species could reproduce quickly during a short window of time when conditions were suitable and would have eventually led to a significant reduction in the mouthparts as the comparatively short-lived adults were freed from seeking food in the arid climate of the Early to Middle Triassic [28]. It is certainly true that today aggregating individuals are obvious targets for natural enemies, including ray-finned fish, bats, birds, frogs, and other insectivorous invertebrates [29], and some of these were certainly present during the Triassic, such as the ray-finned fish, frogs, and some insectivorous invertebrates. At the same time, primitive pterosaurs, which possessed small body sizes, sharp teeth, and well-developed external adductors, were regarded as insectivorous during the Triassic [30]. Trichoptera are, however, tightly linked to water sources, and it is most likely that early swarms were along rivers or streams, as most are today. Accordingly, predators availing themselves of such swarms would have lived in or near such freshwater sources, tending to suggest that such diminutive pterosaurs were unlikely to have been key predators of swarming Trichoptera. Furthermore, most flying predators during the Triassic, including the primitive pterosaurs and dragonflies, were diurnal according to the shape of their scleral rings and orbits [31] as well as comparisons with their coeval relatives [32], while most swarming insects were active during the night; there were notable exceptions. Certainly for the Trichoptera such predators would have been less of a concern, as their swarming began mainly after dusk, and peaked before midnight, and even though some swarming species remain active towards dawn, their activities are always complete before that first light sufficient for aerial predators [4,33].

During the Jurassic and the Cretaceous, the body sizes of most pterosaurs were enlarged, and these species were regarded as the piscivorous forms according to the shape of the skull and gut contents [34,35]. Alternatively, birds appeared during the Late Jurassic, with Enantiornithes dominant by the Cretaceous, which has been reported worldwide, including in mid-Cretaceous Kachin amber [14,36]. The dental patterns and absence of gizzard stones in most specimens indicate that Enantiornithes mainly fed on invertebrates [37,38]. Although birds were the major small-flying predators from the Late Jurassic and Cretaceous [38], they seemingly had little impact on swarming because most Mesozoic birds were also diurnal [31]. The absence of nocturnal flying predators during the Mesozoic would have an advantage for swarming Trichoptera.

A total-evidence tip-dating analysis supports the hypothesis that both Hydroptilidae and Psychomyiidae possessed swarming behavior in the Cretaceous but lost this behavior ∼50–53 million years ago (Ma). A similar pattern is also found in other families, such as Lepidostomatidae and Limnephilidae. This episode of time is critical as it was coeval with the rise of echolocating bats. The earliest flying bat fossil, *Onychonycteris finneyi*, was reported from nearly the same time (52 Ma) [39], while the credibility interval for the rise of bats is about 65 to 52 Ma [40]. The fossil record suggests a global distribution of bats by the early Eocene [39,41–43]. The teeth and gut contents indicate that the earliest bats were significant predators of flying insects [41,44]. The earliest definitive evidence of an echolocating-bat, *Icaronycteris index*, was slightly younger and echolocation perhaps emerged quickly among bats [45]. Laryngeal echolocation aided bats in their detection and capture of prey in nocturnal species [46]. To avoid predation by bats, nocturnal insects evolved refined sound-detecting strategies [47], such as some moth lineages (sister group of Trichoptera), who evolved specialized hearing (tympanal organs) as a defensive mechanism against echolocating bats. Tympanic organs and other sound-detecting organs of similar attunement are not present in extant or fossil Trichoptera, and caddisflies are therefore not equipped to detect bat ultrasound, which is unfortunate as it makes them especially susceptible to the hunting of insectivorous bats. It is well known that swarming insects are a favorite food source for bats [48], because the swarm represents a large and concentrated source of food and swarms are also much easier to detect by ultrasound [49]. In fact, studies on the remains of prey from the stomachs or feces of extant bats show that most species are insectivorous and possess their own behavioral strategy: ‘swarm feeding’ and Trichoptera and Diptera are the most common in gut contents of such bats, indicating that they are significant predators of swarming Trichoptera [50]. This phenomenon shows that the presence of echolocating bats resulted in a significant pressure on swarming Trichoptera, and it is possible that this was a contributing factor to the repeated loss of swarming behavior by various trichopterans during the Eocene, representing a strategic response to bat predation.

Trichoptera diversity appears to have fallen behind that of their sister group, the Lepidoptera, during the Cenozoic, and this may have partly been a result of novel predator pressures such as those imposed by the rise of bats. Meanwhile, coevolution with flowering plants certainly gave Lepidoptera an added boost, furthering the diversity differences between these two insect lineages.

Last, the mix of younger and older individuals within these fossil swarms also indicates that the individuals would have mated relatively soon after emergence and without undergoing a period of adult diapause [51]. Adult diapause is often present in extant species that live in dry environments to avoid mating during the dry season and when water levels are insufficient for developing trichopteran larva [52]. The aggregations of individuals of different ages indicate that the environment of fossil species was sufficiently wet to encourage mating and allowing larvae to live for extended periods. This is consistent with previous studies indicating a tropical rainforest climate in the mid-Cretaceous of Myanmar [53].

## MATERIALS AND METHODS

### Materials

All of the specimens studied in this paper are fossils preserved in Kachin amber from mid-Cretaceous deposits in the Hukawng Valley, Tanai Township, Myitkyina District, Kachin State, northern Myanmar, ∼100 km southwest of the Village of Tanai. The age of Kachin amber is dated by U-Pb to 98.79 ± 0.62 Mya, earliest Cenomanian, mid-Cretaceous [54,55]. All new specimens described herein were acquired by Mr Fangyuan Xia and donated for this study in 2015, thus, in full ethical compliance for the study of Kachin amber [56–58]. All specimens described herein are deposited in the College of Life Sciences, Capital Normal University, Beijing, China. Research on fossils preserved in Kachin amber has a long history. To date more than 1000 species of insects have been described from these deposits [59], dating back more than a century, including Tarachoptera [60], Phthiraptera [61], Lepidoptera [62], etc.

### Optical microscopy and photography

All the pictures were superimposed of Z-Stack using a Nikon SMZ 18. All the line drawings were prepared by using Affinity Designer.

### Geometric morphometric analyses

The morphometric data of the new species, including the data on wings and compound eye were measured under a Nikon DS-Ri2 digital camera system. The detailed data of the new specimens are shown in Table S2 and S3. The data of the wings (Tables S4–S6) were subjected to principal component analysis in PAST 3.0. The scatter diagram of the compound eye was analyzed by IBM SPSS Statistics 26.

### Morphological and molecular datasets

In order to investigate the phylogenetic placement of the new fossil species and trace the changing evolution of the swarm during the evolution of Trichoptera, a combined analysis of 77 trichopteran taxa from 15 families involving morphological data (from fossil and extant taxa) and molecular data (from living taxa) are given. A new trichopteran morphological matrix coded by 30 unordered morphological characters (Table S7) was made. All molecular data, including six molecular sequences (28S and 18S rRNA, nuclear genes EF1a and IDH, protein-coding genes CAD and COI, Table S1), were retrieved from GenBank. Sequences of all six genes were aligned by the MAFFT 7.505 online server with the G-INS-i strategy [63]. The final dataset of the genes includes a total of 9738 aligned base pairs.

### Phylogenetic analysis and ancestral-trait reconstruction of swarming behavior

We analyzed datasets with the Bayesian analysis built by Beast v 2.0 [64]. The HKY model with kappa was used in the analysis of the molecular datasets, and the Lewis MK was used on the analysis of the morphological datasets. Both the analysis of the morphological and molecular datasets was run under the Relaxed Clock Log Normal. Prior distributions on the root of Trichoptera and 32 other nodes were set as uninformative uniform distribution and the divergence times of each node based on the ages of the corresponding trichopteran fossil records. In addition, Tip Dates were used on the four fossil taxa and was set as 99 Ma and its prior was set as the Fossilized Birth Death Model. All priors were left as the default values set by BEAUti [65]. The convergence and effective sampling were assessed using Tracer 1.7.2, with all effective sample sizes >200. The Maximum clade credibility tree was obtained by the TreeAnnotator. The ancestral reconstruction trait analysis of swarming behavior of Trichoptera was reconstructed by Mesquite [66]. The information of the swarm was derived from Müller-Peddinghaus [23] and Nowinszky *et al*. [4,5].

### Online content

Extended morphological information and systematic positions of the five new species; a list of reported fossil species of Psychomyiidae and Hydroptilidae; the morphological data matrix; sequence information including GenBank accession numbers for each species included in the analysis; morphological parameters of *Copulariella ramus* gen. et sp. nov.; morphological parameters of *Palerasnitsynus queqiaoi* sp. nov., *P. qixi* sp. nov., *P. aggregatus* sp. nov., and *P. xiuqiu* sp. nov. (Psychomyiidae); parameters of principal component analysis in Trichoptera; taxa for phylogenetic analysis.

## Supplementary Material

nwae227_Supplemental_File

## Data Availability

All data analyzed in this paper, including the phylogenetic and geometric morphometric analyses, are available as part of the Supplementary Material of this paper. The newly proposed names are registered under the following doi numbers in ZooBank: *Copulariella*—urn: lsid: zoobank.org: act: B515E666-AE8B-4633–878D-CBDD059063C9; *Copulariella ramus*—urn: lsid: zoobank.org: act:85182996-AB55-401F-AD6D-52E99F8AB0D8; *Palerasnitsynus queqiaoi*—urn: lsid: zoobank.org: act: F1DB1F71-11F6-46FD-A870-F02DC7410845; *Palerasnitsynus qixi*—urn: lsid: zoobank.org: act: E22E788C-F711-45F7-A968-79924B4EA885; *Palerasnitsynus xiuqiu*—urn: lsid: zoobank.org: act:92A3AB45-D6E0-4FF3-A103-6A0C191E1791; *Palerasnitsynus aggregatus—*urn: lsid: zoobank.org: act:58E01C1B-FFD7-4366-A1F3-EE5B251A006C.

## CODE AVAILABILITY

The phylogenetic datasets and the commands necessary to run the Beast analyses are included in Tables S1 and S7.
