# Supplementary material for: Swarming caddisflies in the mid-cretaceous
Source: Natl Sci Rev. 2024 Jun 28;11(8):nwae227. doi: 10.1093/nsr/nwae227 (PMC11321250; doi:10.1093/nsr/nwae227)
Supplement: nwae227_Supplemental_File [file nwae227_supplemental_file.doc]

**Supporting Information**

**to**

**Swarming Caddisflies in the Mid-Cretaceous**

Jiajia Wang, Michael S. Engel, Weiting Zhang, Chungkun Shih, Rui Qiu and Dong Ren

Content

[Supporting text 2](#__RefHeading___Toc169877366)

[S1 Systematic Paleontology 2](#__RefHeading___Toc169877367)

[S1.1 New genus of Hydroptilidae 2](#__RefHeading___Toc169877368)

[S1.2 New species of Psychomyiidae 3](#__RefHeading___Toc169877369)

[S1.2.1 *Palerasnitsynus queqiaoi* Wang, Engel, Zhang, Shih et Ren sp. nov. 3](#__RefHeading___Toc169877370)

[S1.2.2 *Palerasnitsynus qixi* Wang, Engel, Zhang, Shih et Ren sp. nov. 4](#__RefHeading___Toc169877371)

[S1.2.3 *Palerasnitsynus aggregatus* Wang, Engel, Zhang, Shih et Ren sp. nov. 4](#__RefHeading___Toc169877372)

[S1.2.4 *Palerasnitsynus xiuqiu* Wang, Engel, Zhang, Shih et Ren sp. nov. 5](#__RefHeading___Toc169877373)

[S2 The Morphological Characters of the Phylogenetic Analysis 6](#__RefHeading___Toc169877374)

[Figures S1 to S8 7](#__RefHeading___Toc169877375)

[Tables S1 to S7 15](#__RefHeading___Toc169877376)

[SI References 40](#__RefHeading___Toc169877377)

**This PDF file includes:**

Supporting text

Figures S1 to S8

Tables S1 to S7

SI References

**Supporting text**

**S1 Systematic Paleontology**

**S1.1 New genus of Hydroptilidae**

***Copulariella ramus* gen. et sp. nov.**

Trichoptera Kirby, 1815

Hydroptilidae Stephens, 1836

Burminoptilinae Botosaneanu, 1981

*Copulariella* Wang, Engel, Zhang, Shih et Ren gen. nov.

**Type species.** *Copulariella ramus* Wang, Engel, Zhang, Shih et Ren sp. nov.

**Diagnosis.** *Copulariella* gen. nov. could be assigned to the subfamily Burminoptilinae of Hydroptilidae based on the following characters: the forewing relatively broad and bearing rounded apices; forks II–IV present in the forewing; Dc open in forewing and hind wing; Cu_1_ sample and unforked. *Copulariella* could be distinguished from *Burminoptila* by the following characters: maxillary palp segment V slightly longer than III or IV of same length, maxillary palp segment I and II short and spur formula 0/2/4. *Copulariella* is different from *Cretacoptila* based on the following characters: mesoscutum covered with a pair of oval setal warts, and the inferior appendage 1-segmented and not forked or not carried black spine, crossveins cu and cu-a contacting at almost the same point on the Cu_2_ and two crossveins drawing a nearly straight line (Fig. 2A-B and Supplementary Fig. S7A).

*Copulariella ramus* Wang, Engel, Zhang, Shih et Ren sp. nov.

**Etymology.** The generic name is derived from the Latin “*cōpulāre*”, which means “couple” or “join”, referring to the aggregation of the specimen. The gender of the name is feminine. The specific epithet is derived from the Latin *rāmus*, which means “branch”, referring to the branching of the intermediate appendage.

**Holotype.** CNU-TRI-MA-2015503, males and females.

**Locality and horizon.** Hukawng Village, Kachin State, northern Myanmar; lowermost Cenomanian, mid-Cretaceous.

**Diagnosis.** As for the genus (*vide supra*).

**S1.2 New species of Psychomyiidae**

Trichoptera Kirby, 1815

Annulipalpia Martynov, 1924

Psychomyiidae Walker, 1852

*Palerasnitsynus* Wichard, Ross et Ross, 2011

**Type species.** *Palerasnitsynus ohlhoffi* Wichard, Ross et Ross, 2011

**Diagosis.** Based on the characters of genus *Palerasnitsynus* Wichard Ross and Ross, 2011

**Included taxa** *P. furcates* Wichard Müller and Wang, 2018, *P. gracilis* Wichard Müller and Wang, 2018, *P. Lepidus* Wichard Müller and Wang, 2018, *P. ohlhoffi* Wichard Ross and Ross, 2011, *P. spinosus* Wichard Müller and Wang, 2018, *P. subglobolus* Wichard Müller and Wang, 2018, *P. subgrandis* Wichard Müller and Wang, 2018, *P. sukatchevae* Wichard Müller and Wang, 2018, *P. vulgaris* Wichard Müller and Wang, 2018. *P. queqiaoi* this paper, *P. qixi* this paper*, P. aggregatus* this paper, *P. xiuqiu* this paper*.*

**S1.2.1 *Palerasnitsynus queqiaoi* Wang, Engel, Zhang, Shih et Ren sp. nov.**

**Etymology.** The specific epithet is derived from the Mandarin “Que Qiao” (Magpie Bridge), a bridge from a Chinese myth. In this myth, a normal cowherd and a female celestial fall in love with each other. But their love violates the regulations of paradise that God could not marry ordinary person. They are only allowed to meet at the seventh lunar month. At that day, a magpie bridge consisting of thousands of magpies is built on the Milky Way, so they can meet with each other. The specific epithet refers to that lots of individuals of both new species and the magpies are aggregated for love.

**Holotype.** CNU-TRI-MA-2015504, males and females.

**Locality and horizon.** Hukawng Village, Kachin State, northern Myanmar; lowermost Cenomanian, mid-Cretaceous.

**Diagnosis.** Superior appendage straight, subequal to the intermediate appendage in the length. The intermediate appendages shorter than superior appendage, divided into outer and inner branches, the outer shorter than the inner in the length, both base of them board and turn dark spine at the apex. Inferior appendages two-segmented, including the coxopodite and harpago. The apex of inferior appendages covered with stout black spines, which distribute around the margin of inferior appendages. The phallic apparatus half the length of Inferior appendage, membranous and broad at the base, the apex bifid and heart-shaped (Supplementary Fig. S7B).

**S1.2.2 *Palerasnitsynus qixi* Wang, Engel, Zhang, Shih et Ren sp. nov.**

**Etymology.** The specific epithet is derived from the Mandarin “Qi Xi”, a Chinese traditional Festival on 7th July, which is known as the Chinese Valentine's Day.

**Holotype.** CNU-TRI-MA-2015505, males.

**Locality and horizon.** Hukawng Village, Kachin State, northern Myanmar; lowermost Cenomanian, mid-Cretaceous.

**Diagnosis.** The superior appendage rod-shaped, covered with long and fine setae and the apex narrow, about half the length of the inferior appendage. Intermediate appendages including two pairs, all branches of intermediate appendages slim and apically forked, subequal in the length and about half the length of the superior appendage. The inferior appendage two-segmented. The harpago more than 5 times as long as the coxopodite. The apex of harpago bearing short black spines. The apexes of two harpago quite close to each other. The phallic apparatus slime and long, bifid with the irregular processes at the apex, covered with the hair setae (Supplementary Fig. S7C).

**S1.2.3 *Palerasnitsynus aggregatus* Wang, Engel, Zhang, Shih et Ren sp. nov.**

**Etymology.** The specific epithet is derived from the Latin “***aggregātus*”** (clustered), referring to the cluster of many individuals of the species in a single piece of amber.

**Holotype.** CNU-TRI-MA-2015506, males and females.

**Paratypes.** CNU-TRI-MA-2015508, males

**Locality and horizon.** Hukawng Village, Kachin State, northern Myanmar; lowermost Cenomanian, mid-Cretaceous.

**Diagnosis.** *Palerasnitsynus aggregatus* sp. nov. can be distinguished from other species most notably by the male genitalia. The superior appendage covered with long and fine setae and the apex narrow, about half length of inferior appendage and subequal to the preanal appendages. The intermediate appendages elongated. In ventral view, both outer and inner margins of intermediate appendage curved, and the outer branch slightly longer than inner branch. The inner branch of intermediate appendage sharp and dark. The inferior appendage two-segmented, coxopodite and harpago, the length of the harpago more than five times as long as coxopodite. The apex of both harpago quite close to each other and bearing short black spines. Phallic apparatus bifid and forming apically two rounded lobes U-shaped (Fig. 7F and Supplementary Fig. S7D).

**S1.2.4 *Palerasnitsynus*** ***xiuqiu* Wang, Engel, Zhang, Shih et Ren sp. nov.**

**Etymology.** The specific epithet is derived from Mandarin “Xiu Qiu” (embroidered ball), a Chinese common mascot made of strips of silk. In traditional Chinese folk custom, when a girl reaches the marriageable age but does not have a boyfriend, she will stand on a high building and her suitors will gather under the building. The girl will choose her love among them and throws the embroidered ball to him. But whoever get the embroidered ball will become the girl’s husband.

**Holotype.** CNU-TRI-MA-2015507, males.

**Locality and horizon.** Hukawng Village, Kachin State, northern Myanmar; lowermost Cenomanian, mid-Cretaceous.

**Diagnosis.** *Palerasnitsynus xiuqiu* sp. nov. could be distinguished from other species by the following characters on the male genitalia. Superior appendage subequal to the intermediate appendage in the length. The intermediate branched at the centre, the outer branch equal to the inner branch in the length. The outer branch stout and covered with the dark spine, its base board and apex narrow. The inner slim and straight. Inferior appendages two-segmented. The apex of inferior appendages covered with stout black spines. The phallic apparatus simple and slim, without processes, longer than superior appendages and shorter than the inferior appendages (Supplementary Fig. S7E).

**S2 The Morphological Characters of the** **Phylogenetic Analysis**

1. **Ocelli.** 0, present; 1, absent.
2. **Occipital setal wart.** 0, long, at or extending beyond eye midline; 1, short, less than eye midline, without anterior taper (oblong to quadrate).
3. **Maxillary palp II and III.** 0, II shorter than III; 1, II subequal to III.
4. **Maxillary palp V.** 0, not elongated; 1, elongated.
5. **Maxillary palp V.** 0, not annulated, flexible or not; 1, annulated and flexible.
6. **Number of maxillary palps in male.** 0, 5; 1, fewer than 5.
7. **Labial palps in female.** 0, with sensilla on terminal segment generally distributed, or if localized, then not forming pad; 1, with large pad of sensilla mesally.
8. **Paralabral sclerites.** 0, not developed; 1, developed.
9. **Labial endite lobes.** 0, absent; 1, present.
10. **Galea and lacinia.** 0, lacinia present as small, distinctly separated structure; 1, lacinia and galea inseparable (forming galeolacinia).
11. **Presence of longitudinal channels on the anterior surface of the haustellum.** 0, absent; 1, present.
12. **Shape of anteclypeolabrum.** 0, short and almost triangular. 1, remarkably elongated ventrally and somewhat rectangular shaped.
13. **Laminatentorium.** 0, small to moderately sized and rather weakly sclerotized; 1, strongly developed; 2, massively developed and strongly sclerotized.
14. **Mandibles.** 0, present as small membranous lobes; 1, absent.
15. **Basalar cleft of anepisternum.** 0, obtuse; 1, acute apically.
16. **Pronotal setal warts.** 0, close to each other; 1, far from each other.
17. **Pronotal setal wart shape.** 0, circle; 1, oval; 2, irregular.
18. **Prostemum with posterior border.** 0, lightly sclerotized medially, dividing the sides of the mesal point into an almost membranous central area; 1, entirely sclerotized and terminating in a narrow mesal point.
19. **Mesoscutum with setal warts.** 0, present; 1, absent.
20. **Mesoscutellar setal wart.** 0, a large round wart; 1, two semicircle warts; 2, two oblong elliptical warts; 3, two oval warts; 4, rounded rectangle wart.
21. **Mesofurca with ventrolateral arm.** 0, tapered to a point; 1, forked.
22. **The terminal of forewing and hind wing.** 0, not very sharp; 1, sharp.
23. **Veins R_2_ and R_3_ in the forewing.** 0, not fused; 1, fused.
24. **Forewing with vein M_4_.** 0, present; 1, absent.
25. **The Mc in the forewing.** 0, present; 1, absent.
26. **Spur formula.** 0, 3/4/4; 1, 1~2/4/4; 2, 1~2/2~3/4; 3, 0~2/2~3/2~3; 4, 0/2~3/4.
27. **The number of spur comb.** 0, 2; 1, 1; 2, 0.
28. **A sclerotized plate on abdominal tergum IX.** 0, absent; 1, present.
29. **Female cerci.** 0, absent; 1, present.
30. **Female with terminal segment of abdomen.** 0, without lateral papillae; 1, with lateral papillae present, subequal in length, and adjacent.

**Figures S1 to S8**

Fig. S1

**
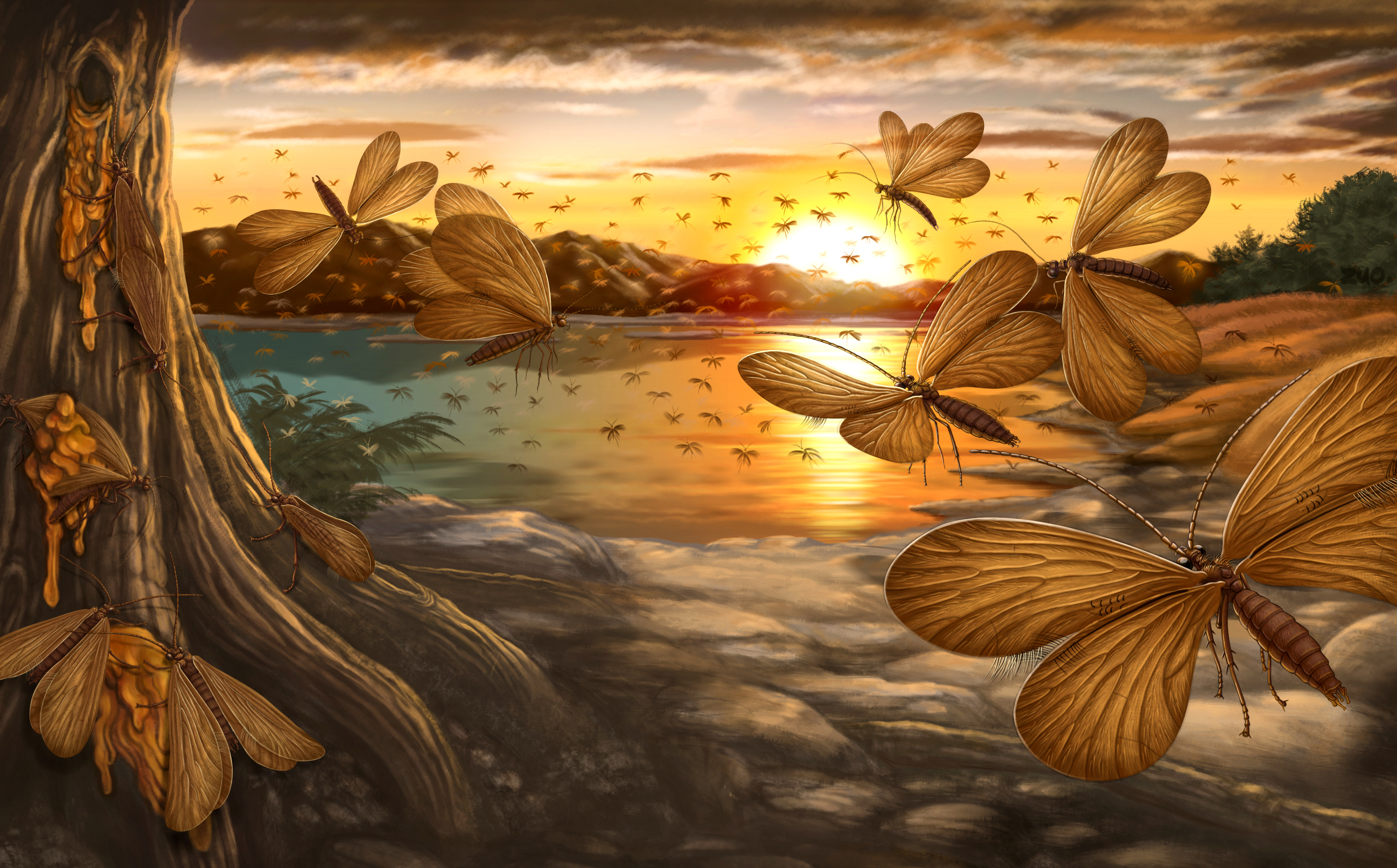
**

**Fig. S1. Ecological reconstruction.**

Ecological reconstruction of *Copulariella ramus* gen. et sp. nov. in Trichoptera. Painted by Xiaoran Zuo.

Fig.S2**

**

**Fig. S2. The swarming caddisflies of new species.** A, *Copulariella* *ramus*, CNU-TRI-MA-2015503. B, *Palerasnitsynus* *queqiaoi*, CNU-TRI-MA-2015504. C, *Palerasnitsynus* *qixi*, CNU-TRI-MA-2015505. D, *Palerasnitsynus* *aggregatus*, CNU-TRI-MA-2015506. Scale bars represent 5000 μm in A–D.

Fig. S3

**
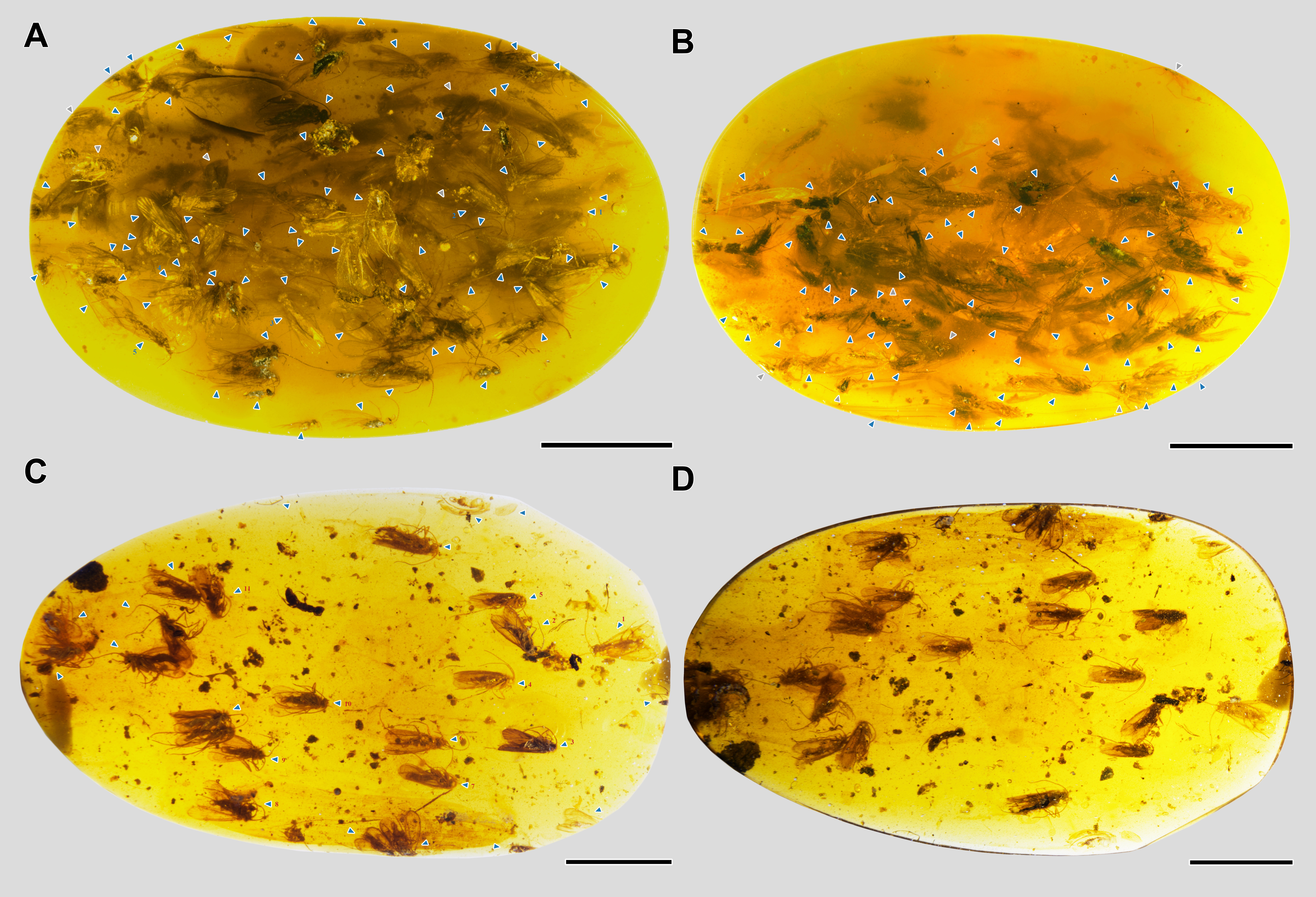
**

**Fig. S3. The swarming caddisflies of new species.** A–B, *Palerasnitsynus* *xiuqiu*, CNU-TRI-MA-2015507. C–D, *Palerasnitsynus* *aggregatus*, CNU-TRI-MA-2015508. Scale bars represent 5000 μm in A–D.

Fig. S4

**
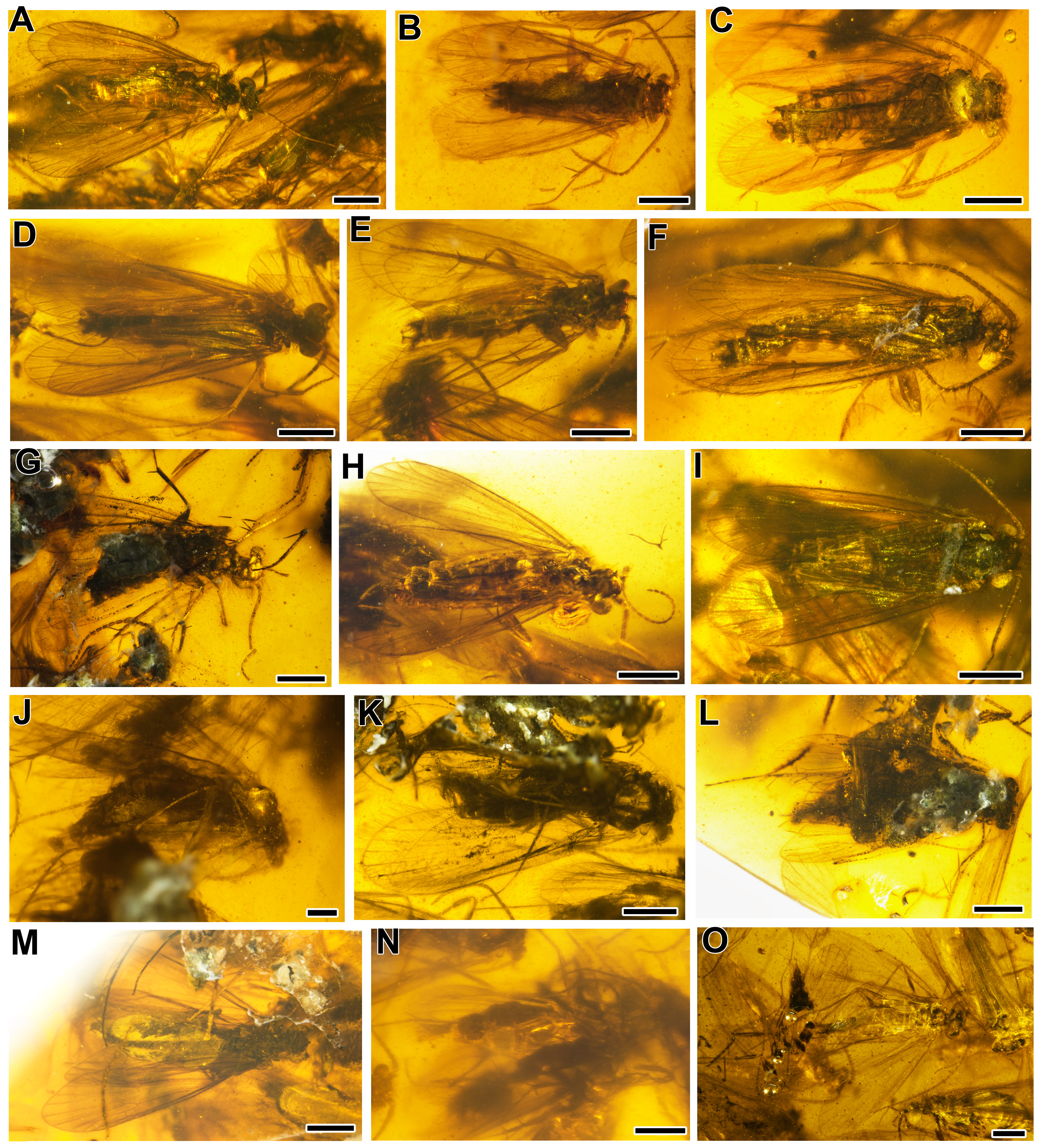
**

**Fig. S4. The origin of the morphological parameters in the individuals.** The origin of the morphological parameters in the individuals from *Copulariella ramus* (A–N, CNU-TRI-MA-2015503) and *Palerasnitsynus queqiaoi* (O, CNU-TRI-MA-2015504). Scale bars represent 500 μm in A–I, K–O and 200 μm in J.

Fig. S5

**
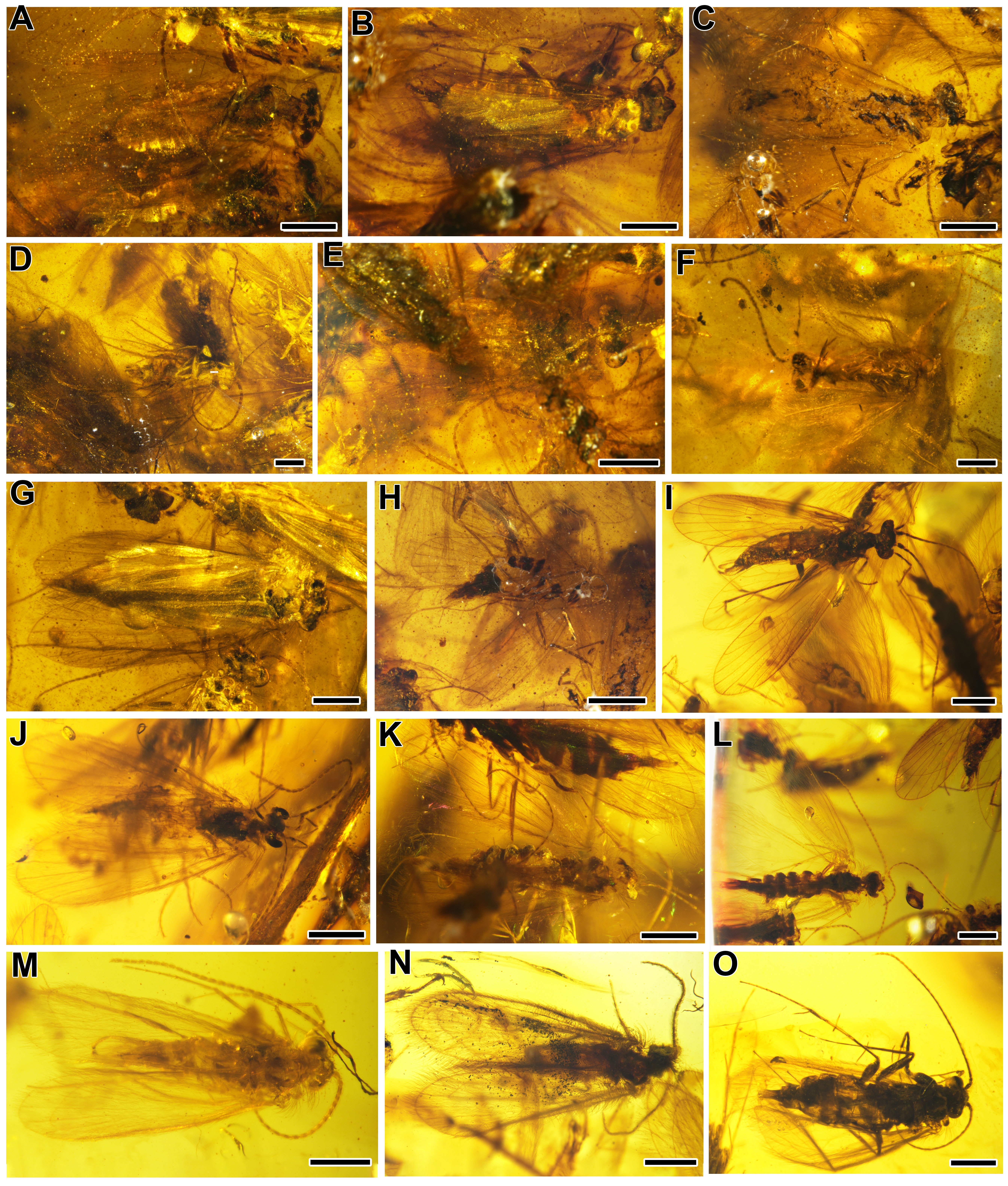
**

**Fig. S5. The origin of the morphological parameters in the individuals.**

The origin of the morphological parameters in the individuals from *Palerasnitsynus queqiaoi* (A–H, CNU-TRI-MA-2015504), P*alerasnitsynus qixi* (I–L, CNU-TRI-MA-2015505) and *Palerasnitsynus aggregatus* (M–O, CNU-TRI-MA-2015506). Scale bars represent 500 μm in A–O.

Fig. S6

**
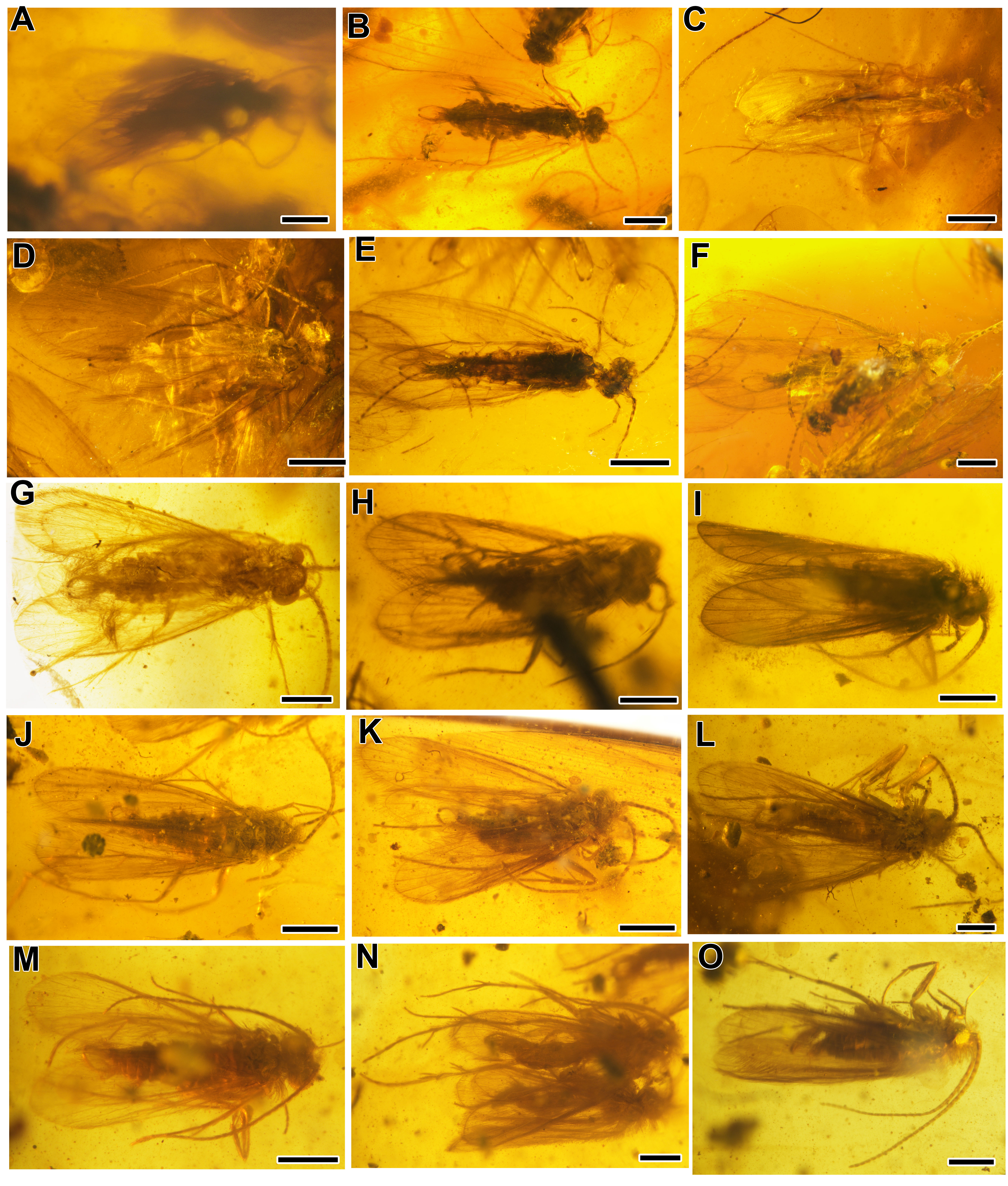
**

**Fig. S6. The origin of the morphological parameters in the individuals.**

The origin of the morphological parameters in the individuals from *Palerasnitsynus xiuqiu* (A–F, CNU-TRI-MA-2015507) and *Palerasnitsynus aggregatus* (G–O, CNU-TRI-MA-2015508). Sacale bars respresent 500 μm in (A–O).

Fig. S7

**

**

**Fig. S7. The male genitalia of swarming caddisflies.**

A, *Copulariella* *ramus*, CNU-TRI-MA-2015503. B, *Palerasnitsynus* *queqiaoi*, CNU-TRI-MA-2015504. C, *Palerasnitsynus* *qixi*, CNU-TRI-MA-2015505. D, *Palerasnitsynus* *aggregatus*, CNU-TRI-MA-2015506. E, *Palerasnitsynus* *xiuqiu*, CNU-TRI-MA-2015507. F, *Palerasnitsynus* *aggregatus*, CNU-TRI-MA-2015508. Scale bars represent 500 μm in A–F.

Fig. S8


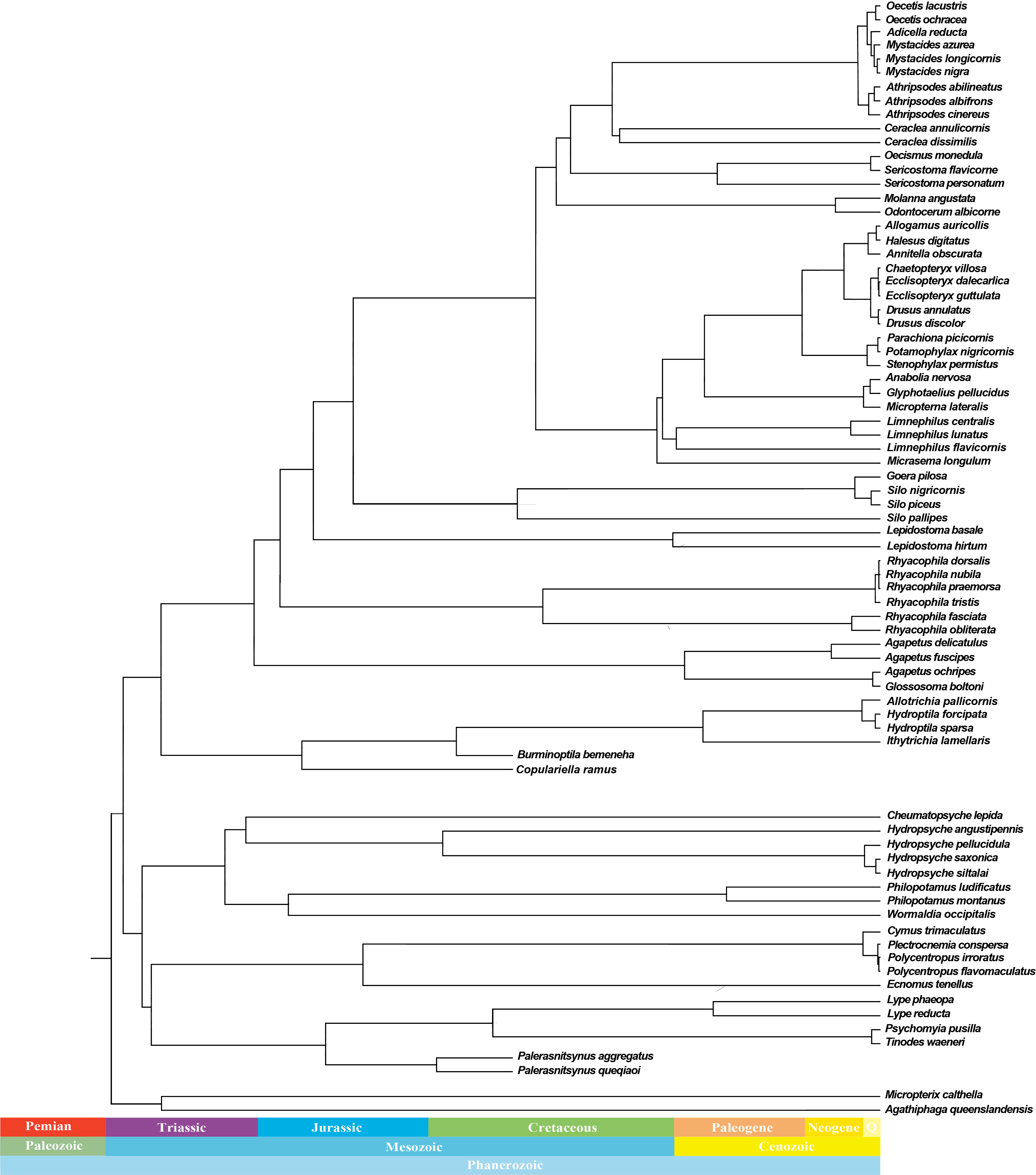


**Fig. S8. Ancestral-trait Reconstruction of Swarming Caddisflies.**

**Tables S1 to S7**

Table S1 The Sequence Information from Genbank Accession Numbers for Each Species Included in the Analysis.

| Suborder | Super-family | Family | Genus | Species | 28S | 18S | CAD | EF1a | IDH | COI |
| --- | --- | --- | --- | --- | --- | --- | --- | --- | --- | --- |
| Zeugloptera (Lepidoptera, outgroup) |  | Micropterigidae | *Micropterix* | *calthella* | MN296519 | MN217255 | KC559453 | GU829241 AF436596 | KC559581 | JF853511 |
| Aglossata (Lepidoptera, outgroup) |  | Agathiphagidae | *Agathiphaga* | *queenslandensis* | MN296520 | AF136884 AF436374 AF136864 | GU828066 | GU828902 | GU829949 | GU695978 |
| Annulipalpia | Philopotamoidea | Hydropsychidae | *Cheumatopsyche* | *lepida* | JQ687965 | JX879413 |  | JQ687894 |  | MW459963 |
| Annulipalpia | Philopotamoidea | Hydropsychidae | *Hydropsyche* | *angustipennis* |  |  | FM998343 | FM998540 |  | HQ569911 |
| Annulipalpia | Philopotamoidea | Hydropsychidae | *Hydropsyche* | *pellucidula* | JQ687935 | JX879415 |  | JQ687871 |  | MW459929 |
| Annulipalpia | Philopotamoidea | Hydropsychidae | *Hydropsyche* | *saxonica* | HM167443 | JX879418 |  |  |  | HQ945700 |
| Annulipalpia | Philopotamoidea | Hydropsychidae | *Hydropsyche* | *siltalai* | JQ687948 | JX879362 | KM225503 | FM998455 | JQ239478 | MW459924 |
| Annulipalpia | Philopotamoidea | Philopotamidae | *Philopotamus* | *ludificatus* |  |  |  |  |  | HM401406 |
| Annulipalpia | Philopotamoidea | Philopotamidae | *Philopotamus* | *montanus* | MN296548 | MN217274 | MN364742 | AF436632 | JQ239504 | JF891239 |
| Annulipalpia | Philopotamoidea | Philopotamidae | *Wormaldia* | *occipitalis* |  |  |  |  |  | JF891238 |
| Annulipalpi | Psychomyioidea | Polycentropodidae | *Cyrnus* | *trimaculatus* | AF436245 | AF436477 | JQ239624 | AF436640 | JQ239483 | MK093958 |
| Annulipalpi | Psychomyioidea | Polycentropodidae | *Plectrocnemia* | *conspersa* |  |  | JQ239633 |  | JQ239490 | MW459925 |
| Annulipalpi | Psychomyioidea | Polycentropodidae | *Polycentropus* | *flavomaculatus* |  |  | JQ239623 |  | JQ239482 | MW459945 |
| Annulipalpi | Psychomyioidea | Polycentropodidae | *Polycentropus* | *irroratus* |  |  |  | FN178815 | JQ239476 | HM401408 |
| Annulipalpi | Psychomyioidea | Psychomyiidae | *Lype* | *phaeopa* | MN296564 | AF436470 | MN364751 | AF436637 |  | HM401395 |
| Annulipalpi | Psychomyioidea | Psychomyiidae | *Lype* | *reducta* |  | JX879399 |  |  |  | GU713198 |
| Annulipalpi | Psychomyioidea | Psychomyiidae | *Psychomyia* | *pusilla* | MN296563 | AF436469, INSbttTHR AAPEI-15 | JQ239701 | INSbttTHR AAPEI-15 | JQ239556 | MW459962 |
| Annulipalpi | Psychomyioidea | Psychomyiidae | *Tinodes* | *waeneri* | AF436354 | AF436467 | FN178940 | AF436636 | JQ239518 | HM401427 |
| Annulipalpi | Psychomyioidea | Ecnomidae | *Ecnomus* | *tenellus* | MN296558 | AF436482 | FN179035 | FN178925 | JQ239549 | HM395763 |
| Spicipalpia |  | Rhyacophilidae | *Rhyacophila* | *dorsalis* |  | JX879306 | MN364857 |  |  | HM862418 |
| Spicipalpia |  | Rhyacophilidae | *Rhyacophila* | *fasciata* |  | JX879308 INSjdsTBSRAAPEI-9 | KM225507 | INSjdsTBSRAAPEI-9 | JQ239524 | HM422071 |
| Spicipalpia |  | Rhyacophilidae | *Rhyacophila* | *nubila* |  |  |  |  |  | MW459960 |
| Spicipalpia |  | Rhyacophilidae | *Rhyacophila* | *obliterata* |  |  |  |  |  | KX296598 |
| Spicipalpia |  | Rhyacophilidae | *Rhyacophila* | *praemorsa* |  |  |  |  |  | GU713167 |
| Spicipalpia |  | Rhyacophilidae | *Rhyacophila* | *tristis* | MN296706 | MN217382 | MN364859 |  |  | HM401417 |
| Spicipalpia |  | Glossosomatidae | *Agapetus* | *delicatulus* |  |  | MN364820 |  |  | KX141965 |
| Spicipalpia |  | Glossosomatidae | *Agapetus* | *fuscipes* |  | JX879411 |  |  |  | HM395973 |
| Spicipalpia |  | Glossosomatidae | *Agapetus* | *ochripes* |  |  |  |  |  | GU713220 |
| Spicipalpia |  | Glossosomatidae | *Glossosoma* | *boltoni* |  |  |  |  |  | HM395656 |
| Spicipalpia |  | Hydroptilidae | *Allotrichia* | *pallicornis* |  |  |  |  |  | GU713201 |
| Spicipalpia |  | Hydroptilidae | *Hydroptila* | *forcipata* |  |  |  |  |  | GU713205 |
| Spicipalpia |  | Hydroptilidae | *Hydroptila* | *sparsa* |  |  |  |  |  | MW459971 |
| Spicipalpia |  | Hydroptilidae | *Ithytrichia* | *lamellaris* |  |  | KM225504 |  | JQ239520 | KX295631 |
| Integripalpia | Leptoceroidea | Leptoceridae | *Adicella* | *reducta* |  | JX879423 | FN601096 | FN600792 | FN601206 | HM395958 |
| Integripalpia | Leptoceroidea | Leptoceridae | *Athripsodes* | *albifrons* |  | JX879376 |  |  |  | MW459851 |
| Integripalpia | Leptoceroidea | Leptoceridae | *Athripsodes* | *bilineatus* |  |  |  |  |  | HM422026 |
| Integripalpia | Leptoceroidea | Leptoceridae | *Athripsodes* | *cinereus* |  | JX879379 | FN601098 | FN600794 | FN601208 | MW459908 |
| Integripalpia | Leptoceroidea | Leptoceridae | *Ceraclea* | *annulicornis* |  |  | KR030393 | KR030353 |  | MZ628951 |
| Integripalpia | Leptoceroidea | Leptoceridae | *Ceraclea* | *dissimilis* | MN296569 | AF436433 | FN601056 | FN600756 | FN601165 | HM395862 |
| Integripalpia | Leptoceroidea | Leptoceridae | *Mystacides* | *azurea* |  | JX879475 |  |  |  | MW459909 |
| Integripalpia | Leptoceroidea | Leptoceridae | *Mystacides* | *longicornis* |  | JX879430 | FN601053 | FN600753 | FN601162 | HM862431 |
| Integripalpia | Leptoceroidea | Leptoceridae | *Mystacides* | *nigra* |  | JX879475 |  |  |  | HM401401 |
| Integripalpia | Leptoceroidea | Leptoceridae | *Oecetis* | *lacustris* | MN296583 | MN217291 | MN364766 | FN600739 | FN601141 | MZ628728 |
| Integripalpia | Leptoceroidea | Leptoceridae | *Oecetis* | *ochracea* |  |  | FN601052 | FN600752 | FN601161 | MG587030 |
| Integripalpia | Leptoceroidea | Molannidae | *Molanna* | *angustata* | MN296579EF417122 | MN217288 | MN364762 | FJ263247 | FN601139 | HM401400 |
| Integripalpia | Leptoceroidea | Odontoceridae | *Odontocerum* | *albicorne* | EF417125 | AF436418 |  |  |  | HQ945698 |
| Integripalpia | Sericostomatoidea | Sericostomatidae | *Oecismus* | *monedula* |  | HG322447 |  |  |  | JF891247 |
| Integripalpia | Sericostomatoidea | Sericostomatidae | *Sericostoma* | *flavicorne* |  |  |  |  |  | KX291874 |
| Integripalpia | Sericostomatoidea | Sericostomatidae | *Sericostoma* | *personatum* |  | JX879403 |  |  |  | HM395956 |
| Integripalpia | Phryganeoidea | Brachycentridae | *Micrasema* | *longulum* |  |  |  |  |  | MW459953 |
| Integripalpia | Phryganeoidea | Lepidostomatidae | *Lepidostoma* | *basale* |  | JX879484 |  |  |  | HQ561936 |
| Integripalpia | Phryganeoidea | Lepidostomatidae | *Lepidostoma* | *hirtum* |  |  | KC559495 |  | KC559640 | MW459835 |
| Integripalpia | Limnphiloidea | Goeridae | *Goera* | *pilosa* | MN296591 | JX879324 |  |  |  | MW459556 |
| Integripalpia | Limnphiloidea | Goeridae | *Silo* | *nigricornis* |  |  | MN364773 |  |  | HM401420 |
| Integripalpia | Limnphiloidea | Goeridae | *Silo* | *pallipes* | MN296594MN296595 | MN217297 | MN364773 |  | KC559630 | HM401423 |
| Integripalpia | Limnphiloidea | Goeridae | *Silo* | *piceus* |  |  |  |  |  | KX295792 |
| Integripalpia | Limnphiloidea | Limnphilidae | *Drusus* | *annulatus* |  | JX879434 |  |  |  | KX296649 |
| Integripalpia | Limnphiloidea | Limnphilidae | *Drusus* | *discolor* |  |  |  |  |  | GU713159 |
| Integripalpia | Limnphiloidea | Limnphilidae | *Ecclisopteryx* | *dalecarlica* | MN296601 | MN217303 | MN364778 |  | KC559643 | KX295414 |
| Integripalpia | Limnphiloidea | Limnphilidae | *Ecclisopteryx* | *guttulata* | MN296601 | MN217303 | MN364778 |  | KC559643 | GU713160 |
|  |  |  |  |  |  |  |  |  |  |  |
| Integripalpia | Limnphiloidea | Limnphilidae | *Anabolia* | *nervosa* |  | JX879389 |  |  |  | MW459399 |
| Integripalpia | Limnphiloidea | Limnphilidae | *Glyphotaelius* | *pellucidus* |  |  |  |  |  | HM401371 |
| Integripalpia | Limnphiloidea | Limnphilidae | *Limnephilus* | *centralis* | MN296603 | KM463951 AF436400 | FN601123 | AF436607 | KF936485 | HM395904 |
| Integripalpia | Limnphiloidea | Limnphilidae | *Limnephilus* | *flavicornis* |  | JX879449 |  |  |  | MZ626698 |
| Integripalpia | Limnphiloidea | Limnphilidae | *Limnephilus* | *lunatus* |  | JX879391 |  |  |  | MZ624034 |
| Integripalpia | Limnphiloidea | Limnphilidae | *Allogamus* | *auricollis* |  |  | MN364775 |  |  | HM395646 |
| Integripalpia | Limnphiloidea | Limnphilidae | *Halesus* | *digitatus* |  |  |  |  |  | MW459773 |
| Integripalpia | Limnphiloidea | Limnphilidae | *Micropterna* | *lateralis* |  |  |  |  |  | MZ626909 |
| Integripalpia | Limnphiloidea | Limnphilidae | *Parachiona* | *picicornis* |  |  |  |  |  | KX291668 |
| Integripalpia | Limnphiloidea | Limnphilidae | *Potamophylax* | *nigricornis* |  | JX879356 |  |  |  | MZ627540 |
| Integripalpia | Limnphiloidea | Limnphilidae | *Stenophylax* | *permistus* |  | JX879470 |  |  |  | KX296282 |
| Integripalpia | Limnphiloidea | Limnphilidae | *Annitella* | *obscurata* |  |  |  | MN606442 |  | MZ625140 |
| Integripalpia | Limnphiloidea | Limnphilidae | *Chaetopteryx* | *villosa* |  | JX879343 |  |  |  | MZ628857 |

Table S2 The morphological parameter of compound eye of new species. A, length of compound eyes; B, width of compound eyes; C, distance between compound eyes.

| Specimen | Number | Gender | A | B | C | B: A | B: C |
| --- | --- | --- | --- | --- | --- | --- | --- |
| *Copulariella ramus*  CNU-TRI-MA-2015503 | 1 | male | 0.18 | 0.10 | 0.35 | 0.56 | 0.29 |
|  | 2 | male | 0.16 | 0.09 | 0.31 | 0.56 | 0.29 |
|  | 3 | male | 0.17 | 0.10 | 0.33 | 0.59 | 0.30 |
|  | 4 | male | 0.21 | 0.11 | 0.29 | 0.52 | 0.38 |
|  | 5 | male | 0.20 | 0.11 | 0.32 | 0.55 | 0.34 |
|  | 6 | male | 0.21 | 0.11 | 0.29 | 0.52 | 0.38 |
|  | 7 | male | 0.19 | 0.10 | 0.29 | 0.53 | 0.34 |
|  | 8 | male | 0.19 | 0.10 | 0.26 | 0.53 | 0.38 |
|  | 9 | male | 0.21 | 0.11 | 0.29 | 0.52 | 0.38 |
|  | 10 | female | 0.17 | 0.13 | 0.33 | 0.76 | 0.39 |
|  | 11 | female | 0.16 | 0.12 | 0.30 | 0.75 | 0.40 |
|  | 12 | female | 0.13 | 0.10 | 0.24 | 0.77 | 0.42 |
|  | 13 | female | 0.17 | 0.13 | 0.31 | 0.76 | 0.42 |
|  | 14 | female | 0.17 | 0.12 | 0.28 | 0.71 | 0.43 |
| *Palerasnitsynus queqiaoi*  CNU-TRI-MA-2015504 | 1 | male | 0.24 | 0.12 | 0.30 | 0.50 | 0.40 |
|  | 2 | male | 0.21 | 0.10 | 0.33 | 0.48 | 0.30 |
|  | 3 | male | 0.22 | 0.14 | 0.28 | 0.64 | 0.50 |
|  | 4 | male | 0.20 | 0.10 | 0.30 | 0.50 | 0.33 |
|  | 5 | male | 0.20 | 0.17 | 0.30 | 0.85 | 0.57 |
|  | 6 | male | 0.22 | 0.11 | 0.29 | 0.50 | 0.38 |
|  | 7 | female | 0.22 | 0.15 | 0.30 | 0.68 | 0.50 |
|  | 8 | female | 0.28 | 0.19 | 0.35 | 0.68 | 0.54 |
|  | 9 | female | 0.22 | 0.15 | 0.33 | 0.68 | 0.45 |
|  | 10 | female | 0.24 | 0.16 | 0.31 | 0.67 | 0.52 |
| *Palerasnitsynus qixi*  CNU-TRI-MA-2015505 | 1 | male | 0.16 | 0.17 | 0.24 | 1.06 | 0.71 |
|  | 2 | male | 0.15 | 0.17 | 0.19 | 1.13 | 0.89 |
|  | 3 | female | 0.17 | 0.10 | 0.24 | 0.59 | 0.42 |
|  | 4 | female | 0.16 | 0.10 | 0.30 | 0.63 | 0.33 |
| *Palerasnitsynus aggregatus*  CNU-TRI-MA-2015506 | 1 | male | 0.21 | 0.18 | 0.25 | 0.86 | 0.72 |
|  | 2 | male | 0.12 | 0.11 | 0.20 | 0.92 | 0.55 |
|  | 3 | female | 0.19 | 0.13 | 0.24 | 0.68 | 0.54 |
|  | 4 | female | 0.18 | 0.11 | 0.26 | 0.61 | 0.42 |

Table S3 The morphological parameter of body and wing of new species. BL, body length; FA, forewing area; FL, forewing length; FW, forewing width; HA, hind wing area; HL, hind wing length; HW, hind wing width; TW, thorax width.

| Species | Number | Gender | BL | FL | FW | FA | HL | HW | HA | TW |
| --- | --- | --- | --- | --- | --- | --- | --- | --- | --- | --- |
| *Copulariella ramus* | 1 | male | 1.86 | 2.19 | 0.68 | 1.22 | 1.82 | 0.58 | 0.79 | 0.32 |
| CNU-TRI-MA-2015503 | 2 | male | 1.73 | 2.03 | 0.65 | 1.00 | 1.78 | 0.54 | 0.71 | 0.34 |
|  | 3 | male | 2.22 | 2.20 | 0.64 | 1.10 | 1.86 | 0.57 | 0.79 | 0.39 |
|  | 4 | male | 2.19 | 3.36 | 0.64 | 1.18 | 1.95 | 0.55 | 0.78 | 0.33 |
|  |  |  |  |  |  |  |  |  |  |  |
|  | 5 | male | 1.87 | 2.14 | 0.69 | 1.10 | 1.71 | 0.55 | 0.69 | 0.28 |
|  | 6 | male | 2.16 | 2.20 | 0.58 | 1.00 | 1.85 | 0.55 | 0.77 | 0.31 |
|  | 7 | male | 1.89 | 2.03 | 0.77 | 1.02 | 1.66 | 0.54 | 0.65 | 0.29 |
|  | 8 | male | 1.86 | 2.05 | 0.66 | 1.01 | 1.59 | 0.58 | 0.66 | 0.59 |
|  | 9 | male | 1.75 | 1.89 | 0.67 | 1.00 | 1.65 | 0.60 | 0.70 | 0.32 |
|  | 10 | male | 1.94 | 2.13 | 0.66 | 1.10 | 1.82 | 0.52 | 0.72 | 0.34 |
|  | 11 | female | 2.23 | 2.48 | 0.79 | 1.45 | 2.03 | 0.63 | 0.90 | 0.41 |
|  | 12 | female | 2.31 | 2.03 | 0.64 | 1.03 | 1.76 | 0.52 | 0.72 | 0.35 |
|  | 13 | female | 2.24 | 2.27 | 0.62 | 1.00 | 1.72 | 0.56 | 0.64 | 0.34 |
|  | 14 | female | 2.43 | 2.30 | 0.65 | ？ | 1.90 | 0.56 | ？ | 0.40 |
| *Palerasnitsynus queqiaoi* | 1 | male | 2.18 | 2.35 | 0.71 | 1.16 | 1.87 | 0.54 | 0.71 | 0.40 |
| CNU-TRI-MA-2015504 | 2 | male | 2.10 | 2.21 | 0.74 | 1.21 | 1.67 | 0.53 | 0.68 | 0.46 |
|  | 3 | male | 2.26 | 2.33 | 0.71 | 1.18 | 1.84 | 0.57 | 0.73 | 0.41 |
|  | 4 | male | 1.85 | 1.92 | 0.64 | 0.93 | 1.62 | 0.51 | 0.64 | 0.40 |
|  | 5 | male | 1.69 | 2.25 | 0.70 | 1.20 | 1.66 | 0.58 | 0.69 | 0.41 |
|  | 6 | male | 1.91 | 1.97 | 0.57 | 0.94 | 1.41 | 0.48 | 0.54 | 0.43 |
|  | 7 | female | 2.03 | 2.02 | 0.62 | 0.91 | 1.80 | 0.55 | 0.64 | 0.44 |
|  | 8 | female | 2.88 | 2.50 | 0.66 | 1.32 | 1.96 | 0.66 | 0.87 | 0.58 |
|  | 9 | female | 2.17 | 2.19 | 0.66 | 1.18 | 1.64 | 0.54 | 0.63 | 0.51 |
| *Palerasnitsynus qixi* | 1 | male | 1.89 | 2.29 | 0.65 | 1.13 | 1.64 | 0.53 | 0.70 | 0.31 |
| CNU-TRI-MA-2015505 | 2 | male | 1.67 | 2.18 | 0.68 | 1.13 | 1.51 | 0.51 | 0.57 | 0.34 |
|  | 3 | female | 1.99 | 2.23 | 0.56 | 1.11 | 1.71 | 0.59 | 0.77 | 0.29 |
|  | 4 | female | 1.97 | 2.01 | 0.70 | 0.98 | 1.49 | 0.46 | 0.55 | 0.34 |
| *Palerasnitsynus aggregatus* | 1 | male | 1.62 | 2.19 | 0.61 | 0.99 | 1.82 | 0.55 | 0.75 | 0.32 |
| CNU-TRI-MA-2015506 | 2 | male | 1.56 | 2.17 | 0.67 | 1.11 | 1.76 | 0.50 | 0.66 | 0.35 |
|  | 3 | female | 2.11 | 2.23 | 0.70 | 1.22 | 1.80 | 0.51 | 0.72 | 0.39 |
| *Palerasnitsynus xiuqiu* | 1 | male | 1.98 | 2.19 | 0.64 | 1.06 | 1.75 | 0.54 | 0.67 | 0.37 |
| CNU-TRI-MA-2015507 | 2 | male | 2.05 | 2.15 | 0.72 | 1.14 | 1.68 | 0.46 | 0.56 | 0.35 |
|  | 3 | male | 2.04 | 2.18 | 0.66 | 0.99 | 1.78 | 0.48 | 0.74 | 0.32 |
|  | 4 | male | 1.95 | 2.19 | 0.68 | 1.19 | 1.83 | 0.58 | 0.76 | 0.35 |
|  | 5 | male | 1.86 | 2.30 | 0.65 | 1.06 | 1.84 | 0.54 | 0.75 | 0.34 |
|  | 6 | male | 2.01 | 2.46 | 0.65 | 1.12 | 1.96 | 0.43 | 0.69 | 0.37 |
| *Palerasnitsynus aggregatus* | 1 | male | 2.06 | 2.46 | 0.72 | 1.26 | 1.97 | 0.5 | 0.76 | 0.41 |
| CNU-TRI-MA-2015508 | 2 | male | 1.73 | 2.08 | 0.67 | 1.04 | 1.61 | 0.49 | 0.61 | 0.42 |
|  | 3 | male | 1.48 | 2.14 | 0.61 | 1.04 | 1.68 | 0.55 | 0.71 | 0.36 |
|  | 4 | male | 1.85 | 2.36 | 0.58 | 0.98 | 1.90 | 0.49 | 0.73 | 0.40 |
|  | 5 | male | 1.75 | 2.58 | 0.64 | 1.27 | 2.00 | 0.53 | 0.78 | 0.39 |
|  | 6 | male | 1.74 | 2.23 | 0.63 | 1.07 | 1.79 | 0.53 | 0.70 | 0.34 |
|  | 7 | male | 1.91 | 2.17 | 0.64 | 1.10 | 1.74 | 0.47 | 0.65 | 0.39 |
|  | 8 | male | 1.89 | 2.32 | 0.62 | 1.16 | 1.88 | 0.54 | 0.78 | 0.40 |
|  | 9 | male | 1.60 | 2.24 | 0.62 | 1.08 | 1.87 | 0.54 | 0.77 | 0.38 |

Table S4 The data of the male of swarming Trichoptera used for parameters of principal component analysis. AR1, (forewing length)/ (wing width); AR2, (wing span) ²/ (total wing area); BL, body length; RWA, (total wing area)/(forewing length); RWL, (forewing length)/(body length); WA, total wing area; WL, wing length; WW, wing width.

| Taxa | swarm(Y/N) | WL | WA | WW | BL | RWL | AR1 | AR2 | RWA |
| --- | --- | --- | --- | --- | --- | --- | --- | --- | --- |
| *Agapetus delicatulus* | N | 4.58 | 16.09 | 2.76 | 3.91 | 1.17 | 1.65 | 4.71 | 3.51 |
| *Agapetus fuscipes* | N | 4.28 | 14.33 | 2.59 | 3.50 | 1.21 | 1.64 | 5.31 | 3.34 |
| *Anabolia nervosa* | N | 14.95 | 237.09 | 12.15 | 10.66 | 1.46 | 1.25 | 4.62 | 15.97 |
| *Annitella obscurata* | N | 12.71 | 221.03 | 12.12 | 11.40 | 1.11 | 1.04 | 4.82 | 17.39 |
| *Allotrichia pallicornis* | N | 5.17 | 15.45 | 2.61 | 3.25 | 1.58 | 1.98 | 6.05 | 2.98 |
| *Cymus trimaculatus* | N | 6.31 | 32.66 | 4.06 | 4.35 | 1.44 | 1.55 | 5.61 | 5.17 |
| *Drusus annulatus* | N | 10.80 | 134.66 | 8.56 | 10.36 | 1.12 | 1.26 | 5.87 | 12.47 |
| *Drusus discolor* | N | 9.25 | 111.70 | 8.44 | 9.20 | 1.01 | 1.09 | 5.96 | 12.08 |
| *Ecclisopteryx dalecarlica* | N | 12.96 | 179.71 | 10.50 | 10.42 | 1.24 | 1.23 | 5.87 | 13.87 |
| *Glyphotaelius pellucidus* | N | 18.20 | 368.08 | 15.38 | 14.61 | 1.30 | 1.18 | 8.02 | 20.22 |
| *Glossosoma pilosa* | N | 11.56 | 132.86 | 9.06 | 8.69 | 1.32 | 1.27 | 8.57 | 11.40 |
| *Hydroptila forcipata* | N | 3.19 | 5.71 | 1.54 | 2.07 | 1.53 | 2.06 | 5.37 | 1.78 |
| *Hydropsyche saxonica* | N | 10.89 | 128.03 | 8.93 | 9.97 | 1.09 | 1.21 | 4.25 | 11.70 |
| *Hydroptila sparsa* | N | 3.13 | 5.29 | 1.54 | 2.69 | 1.16 | 2.03 | 3.88 | 1.69 |
| *Ithytrichia lamellaris* | N | 3.43 | 6.34 | 1.63 | 2.54 | 1.34 | 2.10 | 4.81 | 1.84 |
| *Lype phaeopa* | N | 3.99 | 16.31 | 2.71 | 3.49 | 1.14 | 1.46 | 4.70 | 4.08 |
| *Limnephilus extricatus* | N | 13.48 | 283.51 | 11.64 | 11.60 | 1.16 | 1.16 | 4.22 | 21.03 |
| *Limnephilus fuscicornis* | N | 16.02 | 330.97 | 14.30 | 12.68 | 1.26 | 1.12 | 3.72 | 20.66 |
| *Lepidostoma hirtum* | N | 9.28 | 73.15 | 6.24 | 7.28 | 1.27 | 1.48 | 4.56 | 7.88 |
| *Limnephilus rhombicus* | N | 18.46 | 338.55 | 14.72 | 16.34 | 1.13 | 1.25 | 3.44 | 18.34 |
| *Limnephilus sparsus* | N | 12.43 | 218.64 | 10.86 | 10.61 | 1.17 | 1.14 | 3.43 | 17.58 |
| *Micropterna lateralis* | N | 18.93 | 385.88 | 16.00 | 15.80 | 1.19 | 1.18 | 4.85 | 20.38 |
| *Micrasema longulum* | N | 5.70 | 30.86 | 4.60 | 5.08 | 1.11 | 1.23 | 5.51 | 5.41 |
| *Micrasema setiferum* | N | 5.15 | 21.91 | 3.36 | 3.75 | 1.37 | 1.53 | 5.22 | 4.25 |
| *Potamophylax cingulatus* | N | 21.05 | 520.11 | 18.87 | 15.13 | 1.39 | 1.11 | 6.71 | 24.71 |
| *Plectrocnemia conspersa* | N | 12.13 | 131.88 | 8.88 | 8.79 | 1.37 | 1.36 | 5.18 | 10.80 |
| *Potamophylax latipennis* | N | 21.93 | 482.32 | 17.83 | 15.66 | 1.40 | 1.23 | 1.99 | 21.99 |
| *Potamophylax luctuosus* | N | 21.65 | 428.61 | 16.67 | 18.72 | 1.16 | 1.29 | 5.72 | 19.80 |
| *Parachiona picicornis* | N | 7.20 | 72.53 | 6.02 | 6.32 | 1.21 | 1.19 | 4.20 | 10.07 |
| *Psychomyia pusilla* | N | 5.40 | 18.60 | 2.56 | 4.08 | 1.32 | 2.10 | 5.31 | 3.44 |
| *Rhyacophila dorsalis* | N | 13.46 | 164.01 | 8.83 | 11.31 | 1.19 | 1.52 | 5.03 | 12.10 |
| *Rhyacophila fasciata* | N | 12.35 | 152.25 | 8.80 | 9.42 | 1.31 | 1.40 | 4.43 | 12.30 |
| *Rhyacophila nubila* | N | 12.00 | 129.52 | 7.85 | 10.00 | 1.19 | 1.52 | 5.10 | 10.70 |
| *Rhyacophila obliterata* | N | 12.67 | 163.19 | 9.13 | 9.98 | 1.26 | 1.38 | 5.25 | 12.80 |
| *Rhyacophila praemorsa* | N | 12.61 | 135.55 | 8.25 | 10.68 | 1.18 | 1.52 | 4.53 | 10.70 |
| *Sericostoma flavivorne* | N | 10.47 | 87.52 | 6.87 | 10.78 | 0.97 | 1.52 | 6.39 | 8.35 |
| *Stenophylax permistus* | N | 22.76 | 685.11 | 201.40 | 16.56 | 1.38 | 1.13 | 6.36 | 30.10 |
| *Sericostoma personatum* | N | 11.82 | 121.18 | 7.61 | 10.45 | 1.13 | 1.55 | 5.75 | 10.20 |
| *Tinodes waeneri* | N | 6.99 | 35.15 | 3.90 | 6.31 | 1.10 | 1.79 | 3.58 | 5.02 |
| *Athripsodes albifrons* | Y | 8.29 | 65.95 | 6.02 | 6.56 | 1.26 | 1.37 | 5.77 | 7.95 |
| *Athripsodes bilineatus* | Y | 8.10 | 60.68 | 5.95 | 6.71 | 1.20 | 1.36 | 5.30 | 7.49 |
| *Athripsodes cinereus* | Y | 10.32 | 89.09 | 7.11 | 7.91 | 1.30 | 1.44 | 7.29 | 8.63 |
| *Athripsodes reducta* | Y | 3.77 | 25.86 | 5.44 | 7.36 | 0.51 | 0.69 | 6.51 | 6.85 |
| *Allogamus auricollis* | Y | 9.43 | 114.89 | 8.57 | 9.26 | 1.01 | 1.10 | 5.10 | 12.18 |
| *Agapetus ochripes* | Y | 4.96 | 20.50 | 3.25 | 3.71 | 1.33 | 1.52 | 6.45 | 4.13 |
| *Ceraclea albimacula* | Y | 11.43 | 109.36 | 8.62 | 7.64 | 1.49 | 1.32 | 5.01 | 9.56 |
| *Ceraclea annulicornis* | Y | 10.55 | 100.94 | 7.96 | 7.11 | 1.48 | 1.32 | 5.81 | 9.56 |
| *Ceraclea dissimilis* | Y | 8.18 | 65.40 | 6.31 | 6.64 | 1.23 | 1.29 | 6.53 | 7.99 |
| *Chaetopteryx villosa* | Y | 11.29 | 194.95 | 10.93 | 10.84 | 1.04 | 1.03 | 6.33 | 17.27 |
| *Ecnomus tenellus* | Y | 5.03 | 18.49 | 2.89 | 4.41 | 1.14 | 1.74 | 4.68 | 3.67 |
| *Hydropsyche angustipennis* | Y | 8.44 | 66.85 | 6.09 | 7.44 | 1.13 | 1.38 | 8.67 | 7.92 |
| *Hydropsyche pellucidula* | Y | 12.67 | 143.44 | 9.36 | 9.56 | 1.32 | 1.35 | 4.33 | 11.30 |
| *Hydropsyche siltalai* | Y | 11.81 | 119.99 | 8.57 | 10.48 | 1.12 | 1.37 | 3.11 | 10.10 |
| *Lepidostoma basale* | Y | 10.03 | 73.48 | 5.82 | 8.25 | 1.21 | 1.72 | 3.34 | 7.32 |
| *Limnephilus flavicornis* | Y | 13.76 | 202.01 | 11.02 | 11.61 | 1.18 | 1.25 | 3.58 | 14.68 |
| *Limnephilus lunatus* | Y | 14.13 | 192.36 | 11.03 | 13.15 | 1.07 | 1.28 | 5.04 | 13.61 |
| *Lype reducta* | Y | 2.96 | 15.18 | 4.02 | 5.42 | 0.54 | 0.73 | 4.31 | 5.12 |
| *Mystacides azurea* | Y | 8.03 | 55.49 | 5.41 | 7.15 | 1.12 | 1.48 | 2.82 | 6.91 |
| *Mystacides longicirnis* | Y | 7.27 | 39.05 | 4.54 | 8.75 | 0.82 | 1.60 | 5.30 | 5.37 |
| *Mystacides nigra* | Y | 6.48 | 49.30 | 6.99 | 6.43 | 1.00 | 0.92 | 5.61 | 7.60 |
| *Odontocerum albicorne* | Y | 15.41 | 212.70 | 11.82 | 11.01 | 1.40 | 1.30 | 4.68 | 13.80 |
| *Oecetis lacustris* | Y | 5.41 | 22.22 | 3.12 | 5.13 | 1.05 | 1.73 | 5.44 | 4.10 |
| *Oecismus minedula* | Y | 12.07 | 107.68 | 7.24 | 9.19 | 1.31 | 1.66 | 6.31 | 8.92 |
| *Oecetis ochracea* | Y | 12.59 | 107.65 | 7.13 | 8.99 | 1.40 | 1.76 | 6.23 | 8.55 |
| *Polycentropus flavomaculatus* | Y | 6.51 | 38.45 | 4.37 | 5.66 | 1.15 | 1.49 | 3.77 | 5.90 |
| *Polycentropus irroratus* | Y | 9.38 | 71.60 | 6.18 | 7.10 | 1.31 | 1.51 | 4.70 | 7.63 |
| *Philopotamus ludificatus* | Y | 10.77 | 144.68 | 8.13 | 8.10 | 1.33 | 1.32 | 5.67 | 13.40 |
| *Philopotamus montanus* | Y | 9.89 | 101.57 | 7.44 | 10.05 | 0.98 | 1.32 | 5.22 | 10.20 |
| *Potamophylax nigricornis* | Y | 19.15 | 393.39 | 15.80 | 15.29 | 1.25 | 1.21 | 3.06 | 20.54 |
| *Rhyacophila tristis* | Y | 7.70 | 68.54 | 5.77 | 7.54 | 1.02 | 1.33 | 5.61 | 8.90 |
| *Silo nigricornis* | Y | 9.34 | 76.58 | 7.05 | 6.31 | 1.48 | 1.32 | 3.63 | 8.19 |
| *Silo pallipes* | Y | 7.60 | 53.58 | 5.86 | 6.14 | 1.23 | 1.29 | 3.07 | 7.05 |
| *Silo piceus* | Y | 8.01 | 66.87 | 6.71 | 5.82 | 1.37 | 1.19 | 3.23 | 8.34 |
| *Wormaldia occipitalis* | Y | 4.45 | 51.71 | 7.08 | 8.01 | 0.55 | 0.62 | 5.85 | 11.60 |
| *Copulariella ramus* | Y | 2.22 | 3.60 | 1.22 | 1.95 | 1.14 | 1.82 | 6.38 | 1.62 |
| *Palerasnitsynus agg* | Y | 2.27 | 3.64 | 1.15 | 1.74 | 1.30 | 1.96 | 6.64 | 1.60 |
| *Palerasnitsynus qixi* | Y | 2.24 | 3.53 | 1.19 | 1.78 | 1.26 | 1.88 | 6.51 | 1.58 |
| *Palerasnitsynus queqiao* | Y | 2.17 | 3.54 | 1.21 | 2.00 | 1.09 | 1.79 | 6.41 | 1.63 |
| *Palerasnitsynus xiuqiu* | Y | 2.25 | 3.58 | 1.17 | 1.98 | 1.14 | 1.92 | 6.57 | 1.59 |

Table S5 The data of the female of swarming Trichoptera used for parameters of principal component analysis. FAR1, (forewing length of female)/ (wing width of female); FAR2, (wing span of female) ²/ (total wing area of female); FBL, body length of female; RWA, (total wing area of female)/(forewing length of female); RWL, (forewing length of female)/(body length of female); FWA, total wing area of female; FWL, wing length of female; FWW, wing width of female.

| Taxa | swarm(Y/N) | FWL | FWA | FWW | FBL | FRWL | FAR1 | FAR2 | FRWA |
| --- | --- | --- | --- | --- | --- | --- | --- | --- | --- |
| *Agapetus delicatulus* | N | 6.52 | 32.32 | 3.75 | 4.37 | 1.49 | 1.74 | 6.28 | 4.95 |
| *Agapetus fuscipes* | N | 4.53 | 15.54 | 2.67 | 4.02 | 1.12 | 1.69 | 6.56 | 3.43 |
| *Anabolia nervosa* | N | 14.17 | 216.72 | 11.60 | 9.87 | 1.44 | 1.22 | 4.27 | 15.29 |
| *Annitella obscurata* | N | 11.12 | 172.51 | 10.58 | 8.82 | 1.27 | 1.05 | 3.35 | 15.51 |
| *Allotrichia pallicornis* | N | 5.30 | 14.73 | 2.40 | 3.79 | 1.40 | 2.20 | 8.85 | 2.77 |
| *Cymus trimaculatus* | N | 7.49 | 44.65 | 4.62 | 5.28 | 1.41 | 1.62 | 5.99 | 5.96 |
| *Drusus annulatus* | N | 9.51 | 95.67 | 7.48 | 7.01 | 1.37 | 1.27 | 4.42 | 10.06 |
| *Drusus discolor* | N | 8.87 | 101.32 | 8.00 | 8.23 | 1.07 | 1.10 | 3.68 | 11.43 |
| *Ecclisopteryx dalecarlica* | N | 12.31 | 159.86 | 9.87 | 9.78 | 1.26 | 1.24 | 4.43 | 12.99 |
| *Glyphotaelius pellucidus* | N | 16.40 | 355.67 | 13.92 | 12.84 | 1.28 | 1.23 | 3.99 | 21.68 |
| *Glossosoma pilosa* | N | 12.26 | 156.70 | 9.82 | 9.82 | 1.24 | 1.24 | 4.61 | 12.70 |
| *Hydroptila forcipata* | N | 3.61 | 7.46 | 1.90 | 3.24 | 1.11 | 1.90 | 8.55 | 2.77 |
| *Hydropsyche saxonica* | N | 12.88 | 133.05 | 8.57 | 10.48 | 1.26 | 1.50 | 5.89 | 10.30 |
| *Hydroptila sparsa* | N | 3.09 | 5.81 | 1.51 | 3.13 | 0.98 | 2.05 | 7.85 | 1.88 |
| *Ithytrichia lamellaris* | N | 3.33 | 5.94 | 1.53 | 2.81 | 1.18 | 2.17 | 8.82 | 1.78 |
| *Lype phaeopa* | N | 4.98 | 21.88 | 3.05 | 4.53 | 1.09 | 1.62 | 5.99 | 4.39 |
| *Limnephilus extricatus* | N | 11.78 | 190.96 | 10.41 | 9.08 | 1.30 | 1.13 | 3.39 | 16.21 |
| *Limnephilus fuscicornis* | N | 11.47 | 184.05 | 10.08 | 9.24 | 1.24 | 1.13 | 3.37 | 16.05 |
| *Lepidostoma hirtum* | N | 10.68 | 90.90 | 6.79 | 5.89 | 1.81 | 1.57 | 5.98 | 8.51 |
| *Limnephilus rhombicus* | N | 19.04 | 352.85 | 14.95 | 15.01 | 1.26 | 1.27 | 4.78 | 18.53 |
| *Limnephilus sparsus* | N | 11.70 | 187.07 | 10.12 | 9.09 | 1.28 | 1.15 | 3.42 | 15.99 |
| *Micropterna lateralis* | N | 18.27 | 355.03 | 15.24 | 14.01 | 1.30 | 1.19 | 4.27 | 19.43 |
| *Micrasema longulum* | N | 5.55 | 38.15 | 6.14 | 4.42 | 1.25 | 0.90 | 3.86 | 6.87 |
| *Micrasema setiferum* | N | 5.26 | 21.20 | 3.39 | 3.94 | 1.33 | 1.55 | 6.36 | 4.03 |
| *Potamophylax cingulatus* | N | 20.52 | 435.64 | 16.91 | 13.98 | 1.47 | 1.21 | 4.39 | 21.23 |
| *Plectrocnemia conspersa* | N | 12.46 | 157.96 | 9.69 | 10.65 | 1.16 | 1.28 | 4.69 | 12.60 |
| *Potamophylax latipennis* | N | 20.23 | 415.94 | 16.61 | 14.75 | 1.37 | 1.21 | 4.47 | 20.56 |
| *Potamophylax luctuosus* | N | 20.48 | 366.87 | 16.60 | 14.78 | 1.39 | 1.23 | 4.23 | 17.91 |
| *Parachiona picicornis* | N | 8.68 | 88.16 | 7.43 | 6.52 | 1.33 | 1.17 | 3.99 | 10.16 |
| *Psychomyia pusilla* | N | 5.95 | 21.31 | 2.60 | 4.95 | 1.19 | 2.27 | 7.60 | 3.58 |
| *Rhyacophila dorsalis* | N | 13.16 | 157.80 | 8.77 | 11.31 | 1.16 | 1.49 | 5.07 | 11.90 |
| *Rhyacophila fasciata* | N | 12.15 | 150.13 | 8.88 | 9.49 | 1.28 | 1.36 | 4.57 | 12.30 |
| *Rhyacophila nubila* | N | 12.83 | 151.07 | 8.55 | 10.99 | 1.16 | 1.50 | 5.16 | 11.70 |
| *Rhyacophila obliterata* | N | 10.85 | 112.23 | 7.12 | 9.28 | 1.17 | 1.52 | 5.05 | 10.30 |
| *Rhyacophila praemorsa* | N | 11.32 | 113.96 | 7.36 | 10.47 | 1.08 | 1.53 | 4.92 | 10.00 |
| *Sericostoma flavivorne* | N | 11.91 | 107.89 | 7.54 | 10.63 | 1.12 | 1.58 | 6.29 | 9.05 |
| *Stenophylax permistus* | N | 22.17 | 638.87 | 19.58 | 14.98 | 1.49 | 1.13 | 3.50 | 28.82 |
| *Sericostoma personatum* | N | 13.56 | 135.73 | 8.00 | 10.39 | 1.30 | 1.69 | 6.45 | 10.00 |
| *Tinodes waeneri* | N | 7.18 | 39.28 | 4.35 | 6.56 | 1.09 | 1.65 | 6.50 | 5.47 |
| *Athripsodes albifrons* | Y | 7.51 | 51.93 | 5.19 | 6.37 | 1.17 | 1.44 | 5.11 | 6.91 |
| *Athripsodes bilineatus* | Y | 7.49 | 52.46 | 5.27 | 6.89 | 1.08 | 1.41 | 5.01 | 7.00 |
| *Athripsodes cinereus* | Y | 9.62 | 74.62 | 6.26 | 7.41 | 1.29 | 1.53 | 5.75 | 7.75 |
| *Athripsodes reducta* | Y | 3.86 | 27.49 | 5.65 | 5.71 | 0.67 | 0.68 | 2.75 | 7.12 |
| *Allogamus auricollis* | Y | 10.07 | 120.34 | 8.77 | 9.02 | 1.12 | 1.14 | 3.95 | 11.95 |
| *Agapetus ochripes* | Y | 5.07 | 19.96 | 3.12 | 4.00 | 1.26 | 1.62 | 6.33 | 3.93 |
| *Ceraclea albimacula* | Y | 9.38 | 87.19 | 7.78 | 7.11 | 1.32 | 1.20 | 4.83 | 7.83 |
| *Ceraclea annulicornis* | Y | 6.72 | 52.64 | 5.46 | 6.45 | 1.04 | 1.22 | 4.20 | 7.83 |
| *Ceraclea dissimilis* | Y | 4.65 | 44.42 | 7.36 | 6.18 | 0.75 | 0.63 | 2.77 | 9.55 |
| *Chaetopteryx villosa* | Y | 9.69 | 125.04 | 9.27 | 8.45 | 1.14 | 1.04 | 3.65 | 12.90 |
| *Ecnomus tenellus* | Y | 5.68 | 22.56 | 3.10 | 4.70 | 1.20 | 1.83 | 6.80 | 3.97 |
| *Hydropsyche angustipennis* | Y | 10.38 | 95.19 | 7.04 | 8.59 | 1.20 | 1.47 | 5.37 | 9.17 |
| *Hydropsyche pellucidula* | Y | 13.40 | 157.60 | 9.44 | 10.66 | 1.25 | 1.41 | 5.37 | 9.17 |
| *Hydropsyche siltalai* | Y | 10.38 | 103.30 | 7.56 | 10.21 | 1.01 | 1.37 | 5.12 | 9.95 |
| *Lepidostoma basale* | Y | 10.34 | 75.42 | 5.77 | 5.45 | 1.89 | 1.79 | 6.54 | 7.29 |
| *Limnephilus flavicornis* | Y | 17.17 | 352.12 | 14.75 | 13.85 | 1.24 | 1.16 | 3.88 | 20.51 |
| *Limnephilus lunatus* | Y | 14.34 | 190.54 | 11.03 | 13.15 | 1.18 | 1.01 | 2.91 | 13.29 |
| *Lype reducta* | Y | 4.78 | 22.81 | 3.81 | 4.74 | 1.00 | 1.25 | 5.32 | 4.77 |
| *Mystacides azurea* | Y | 7.49 | 45.59 | 4.72 | 6.90 | 1.08 | 1.58 | 5.77 | 6.08 |
| *Mystacides longicirnis* | Y | 6.97 | 35.21 | 4.15 | 8.08 | 0.86 | 1.67 | 6.42 | 5.05 |
| *Mystacides nigra* | Y | 8.62 | 52.05 | 5.17 | 8.25 | 1.04 | 1.66 | 6.69 | 6.03 |
| *Odontocerum albicorne* | Y | 21.12 | 322.84 | 12.92 | 12.84 | 1.64 | 1.63 | 6.29 | 15.20 |
| *Oecetis lacustris* | Y | 7.28 | 33.24 | 3.72 | 5.57 | 1.30 | 1.95 | 7.39 | 4.56 |
| *Oecismus minedula* | Y | 13.90 | 144.58 | 8.12 | 10.88 | 1.28 | 1.72 | 6.44 | 10.40 |
| *Oecetis ochracea* | Y | 12.76 | 110.62 | 7.30 | 8.75 | 1.45 | 1.74 | 6.72 | 8.66 |
| *Polycentropus flavomaculatus* | Y | 8.24 | 59.71 | 5.35 | 6.69 | 1.23 | 1.54 | 5.60 | 7.24 |
| *Polycentropus irroratus* | Y | 10.78 | 106.25 | 7.68 | 6.91 | 1.56 | 1.40 | 5.34 | 9.85 |
| *Philopotamus ludificatus* | Y | 12.25 | 172.30 | 8.84 | 9.24 | 1.32 | 1.38 | 4.09 | 14.00 |
| *Philopotamus montanus* | Y | 11.33 | 124.76 | 8.01 | 10.13 | 1.11 | 1.41 | 4.90 | 11.00 |
| *Potamophylax nigricornis* | Y | 18.88 | 426.25 | 15.29 | 11.63 | 1.64 | 1.20 | 4.31 | 22.58 |
| *Rhyacophila tristis* | Y | 8.62 | 67.29 | 5.73 | 9.10 | 0.94 | 1.50 | 5.29 | 7.80 |
| *Silo nigricornis* | Y | 9.13 | 91.96 | 7.60 | 8.48 | 1.07 | 1.20 | 4.33 | 10.00 |
| *Silo pallipes* | Y | 8.89 | 76.93 | 6.82 | 8.91 | 0.99 | 1.30 | 4.91 | 8.65 |
| *Silo piceus* | Y | 8.00 | 72.39 | 6.81 | 7.21 | 1.11 | 1.17 | 4.19 | 9.04 |
| *Wormaldia occipitalis* | Y | 4.45 | 54.42 | 7.41 | 8.70 | 0.51 | 0.60 | 1.92 | 12.20 |
| *Copulariella ramus* | Y | 2.27 | 3.82 | 1.24 | 2.30 | 0.99 | 1.83 | 6.32 | 1.68 |
| *Palerasnitsynus agg* | Y | 2.23 | 3.88 | 1.21 | 2.11 | 1.06 | 1.84 | 6.06 | 1.74 |
| *Palerasnitsynus qixi* | Y | 2.12 | 3.42 | 1.16 | 1.98 | 1.07 | 1.83 | 6.08 | 1.61 |
| *Palerasnitsynus queqiao* | Y | 2.24 | 3.70 | 1.23 | 2.36 | 0.95 | 1.82 | 6.73 | 1.65 |
| *Palerasnitsynus xiuqiu* | Y |  |  |  |  |  |  |  |  |

Table S6 The data of ratio between the measurements of male and female of swarming Trichoptera used for parameters of principal component analysis. WAM/F, (total wing area of male) / (total wing area of female); WLM/F, (wing width of male) / (wing length of female); WWM/F, (wing width of male) / (wing width of female).

| Taxa | swarm(Y/N) | WLM/F | WAM/F | WWM/F |
| --- | --- | --- | --- | --- |
| *Agapetus delicatulus* | N | 0.70 | 0.50 | 0.74 |
| *Agapetus fuscipes* | N | 0.94 | 0.92 | 0.97 |
| *Anabolia nervosa* | N | 1.06 | 1.09 | 1.05 |
| *Annitella obscurata* | N | 1.14 | 1.28 | 1.15 |
| *Allotrichia pallicornis* | N | 0.98 | 1.05 | 1.09 |
| *Cymus trimaculatus* | N | 0.84 | 0.73 | 0.88 |
| *Drusus annulatus* | N | 1.14 | 1.41 | 1.14 |
| *Drusus discolor* | N | 1.04 | 1.10 | 1.06 |
| *Ecclisopteryx dalecarlica* | N | 1.05 | 1.12 | 1.06 |
| *Glyphotaelius pellucidus* | N | 1.11 | 1.03 | 1.10 |
| *Glossosoma pilosa* | N | 0.94 | 0.85 | 0.92 |
| *Hydroptila forcipata* | N | 0.88 | 0.77 | 0.81 |
| *Hydropsyche saxonica* | N | 0.85 | 0.96 | 1.04 |
| *Hydroptila sparsa* | N | 1.01 | 0.91 | 1.02 |
| *Ithytrichia lamellaris* | N | 1.03 | 1.07 | 1.07 |
| *Lype phaeopa* | N | 0.80 | 0.75 | 0.89 |
| *Limnephilus extricatus* | N | 1.14 | 1.48 | 1.12 |
| *Limnephilus fuscicornis* | N | 1.40 | 1.80 | 1.42 |
| *Lepidostoma hirtum* | N | 0.87 | 0.80 | 0.92 |
| *Limnephilus rhombicus* | N | 0.97 | 0.96 | 0.98 |
| *Limnephilus sparsus* | N | 1.06 | 1.17 | 1.07 |
| *Micropterna lateralis* | N | 1.04 | 1.09 | 1.05 |
| *Micrasema longulum* | N | 1.03 | 0.81 | 0.75 |
| *Micrasema setiferum* | N | 0.98 | 1.03 | 0.99 |
| *Potamophylax cingulatus* | N | 1.03 | 1.19 | 1.12 |
| *Plectrocnemia conspersa* | N | 0.97 | 0.83 | 0.92 |
| *Potamophylax latipennis* | N | 1.08 | 1.16 | 1.07 |
| *Potamophylax luctuosus* | N | 1.06 | 1.17 | 1.00 |
| *Parachiona picicornis* | N | 0.83 | 0.82 | 0.81 |
| *Psychomyia pusilla* | N | 0.91 | 0.87 | 0.98 |
| *Rhyacophila dorsalis* | N | 1.02 | 1.04 | 1.01 |
| *Rhyacophila fasciata* | N | 1.02 | 1.01 | 0.99 |
| *Rhyacophila nubila* | N | 0.94 | 0.86 | 0.92 |
| *Rhyacophila obliterata* | N | 1.17 | 1.45 | 1.28 |
| *Rhyacophila praemorsa* | N | 1.11 | 1.19 | 1.12 |
| *Sericostoma flavivorne* | N | 0.88 | 0.81 | 0.91 |
| *Stenophylax permistus* | N | 1.03 | 1.07 | 10.29 |
| *Sericostoma personatum* | N | 0.87 | 0.89 | 0.95 |
| *Tinodes waeneri* | N | 0.97 | 0.89 | 0.90 |
| *Athripsodes albifrons* | Y | 1.10 | 1.27 | 1.16 |
| *Athripsodes bilineatus* | Y | 1.08 | 1.16 | 1.13 |
| *Athripsodes cinereus* | Y | 1.07 | 1.19 | 1.14 |
| *Athripsodes reducta* | Y | 0.98 | 0.94 | 0.96 |
| *Allogamus auricollis* | Y | 0.94 | 0.95 | 0.98 |
| *Agapetus ochripes* | Y | 0.98 | 1.03 | 1.04 |
| *Ceraclea albimacula* | Y | 1.22 | 1.25 | 1.11 |
| *Ceraclea annulicornis* | Y | 1.57 | 1.92 | 1.46 |
| *Ceraclea dissimilis* | Y | 1.76 | 1.47 | 0.86 |
| *Chaetopteryx villosa* | Y | 1.17 | 1.56 | 1.18 |
| *Ecnomus tenellus* | Y | 0.89 | 0.82 | 0.93 |
| *Hydropsyche angustipennis* | Y | 0.81 | 0.70 | 0.87 |
| *Hydropsyche pellucidula* | Y | 0.95 | 0.91 | 0.99 |
| *Hydropsyche siltalai* | Y | 1.14 | 1.16 | 1.13 |
| *Lepidostoma basale* | Y | 0.97 | 0.97 | 1.01 |
| *Limnephilus flavicornis* | Y | 0.80 | 0.57 | 0.75 |
| *Limnephilus lunatus* | Y | 0.99 | 1.01 | 1.00 |
| *Lype reducta* | Y | 0.62 | 0.67 | 1.06 |
| *Mystacides azurea* | Y | 1.07 | 1.22 | 1.15 |
| *Mystacides longicirnis* | Y | 1.04 | 1.11 | 1.09 |
| *Mystacides nigra* | Y | 0.75 | 0.95 | 1.35 |
| *Odontocerum albicorne* | Y | 0.73 | 0.66 | 0.91 |
| *Oecetis lacustris* | Y | 0.74 | 0.67 | 0.84 |
| *Oecismus minedula* | Y | 0.87 | 0.74 | 0.89 |
| *Oecetis ochracea* | Y | 0.99 | 0.97 | 0.98 |
| *Polycentropus flavomaculatus* | Y | 0.79 | 0.64 | 0.82 |
| *Polycentropus irroratus* | Y | 0.87 | 0.67 | 0.80 |
| *Philopotamus ludificatus* | Y | 0.88 | 0.84 | 0.92 |
| *Philopotamus montanus* | Y | 0.87 | 0.81 | 0.93 |
| *Potamophylax nigricornis* | Y | 1.01 | 0.92 | 1.03 |
| *Rhyacophila tristis* | Y | 0.89 | 1.02 | 1.01 |
| *Silo nigricornis* | Y | 1.02 | 0.83 | 0.93 |
| *Silo pallipes* | Y | 0.85 | 0.70 | 0.86 |
| *Silo piceus* | Y | 1.00 | 0.92 | 0.99 |
| *Wormaldia occipitalis* | Y | 1.00 | 0.95 | 0.96 |
| *Copulariella ramus* | Y | 0.98 | 0.94 | 0.98 |
| *Palerasnitsynus agg* | Y | 1.02 | 0.94 | 0.95 |
| *Palerasnitsynus qixi* | Y | 1.05 | 1.03 | 1.03 |
| *Palerasnitsynus queqiao* | Y | 0.97 | 0.96 | 0.98 |
| *Palerasnitsynus xiuqiu* | Y | WLM/F | WAM/F | WWM/F |

Table S7 Character matrix of 75 taxa used for phylogenetic analysis

| Number | 1 | 2 | 3 | 4 | 5 | 6 | 7 | 8 | 9 | 10 | 11 | 12 |
| --- | --- | --- | --- | --- | --- | --- | --- | --- | --- | --- | --- | --- |
| *Cheumatopsyche lepida* | 1 | 0 | 0 | 1 | 1 | 0 | 0 | 0 | 1 | 0 | 0 | 0 |
| *Hydropsyche angustipennis* | 1 | 0 | 0 | 1 | 1 | 0 | 0 | 0 | 1 | 0 | 0 | 0 |
| *Hydropsyche pellucidula* | 1 | 0 | 0 | 1 | 1 | 0 | 0 | 0 | 1 | 0 | 0 | 0 |
| *Hydropsyche saxonica* | 1 | 0 | 0 | 1 | 1 | 0 | 0 | 0 | 1 | 0 | 0 | 0 |
| *Hydropsyche siltalai* | 1 | 0 | 0 | 1 | 1 | 0 | 0 | 0 | 1 | 0 | 0 | 0 |
| *Philopotamus ludificatus* | 0 | 0 | 0 | 1 | 1 | 0 | 0 | 0 | 1 | 0 | 0 | 0 |
| *Philopotamus montanus* | 0 | 0 | 0 | 1 | 1 | 0 | 0 | 0 | 1 | 0 | 0 | 0 |
| *Wormaldia occipitalis* | 0 | 0 | 0 | 1 | 1 | 0 | 0 | 0 | 1 | 0 | 0 | 0 |
| *Cymus trimaculatus* | 0 | 1 | 0 | 1 | 1 | 0 | 0 | 0 | 1 | 0 | 1 | 0 |
| *Plectrocnemia conspersa* | 0 | 1 | 0 | 1 | 1 | 0 | 0 | 0 | 1 | 0 | 1 | 0 |
| *Polycentropus flavomaculatus* | 0 | 1 | 0 | 1 | 1 | 0 | 0 | 0 | 1 | 0 | 1 | 0 |
| *Polycentropus irroratus* | 0 | 1 | 0 | 1 | 1 | 0 | 0 | 0 | 1 | 0 | 0 | 0 |
| *Lype phaeopa* | 1 | 0 | 0 | 1 | 1 | 0 | 0 | 0 | 1 | 0 | 0 | 0 |
| *Lype reducta* | 1 | 0 | 0 | 1 | 1 | 0 | 0 | 0 | 1 | 0 | 0 | 0 |
| *Palerasnitsynus queqiaoi* | 1 | 0 | 0 | 1 | 1 | 0 | 0 | 0 | 1 | 0 | 0 | 0 |
| *Palerasnitsynus qixi* | 1 | 0 | 0 | 1 | 1 | 0 | 0 | 0 | 1 | 0 | 0 | 0 |
| *Psychomyia pusilla* | 1 | 0 | 0 | 1 | 1 | 0 | 0 | 0 | 1 | 0 | 0 | 0 |
| *Tinodes waeneri* | 1 | 0 | 0 | 1 | 1 | 0 | 0 | 0 | 1 | 0 | 0 | 0 |
| *Ecnomus tenellus* | 1 | 0 | 0 | 1 | 1 | 0 | 0 | 0 | 1 | 0 | 0 | 0 |
| *Rhyacophila dorsalis* | 0 | 0 | 0 | 0 | 0 | 0 | 0 | 0 | 1 | 0 | 0 | 0 |
| *Rhyacophila fasciata* | 0 | 0 | 0 | 0 | 0 | 0 | 0 | 0 | 1 | 0 | 0 | 0 |
| *Rhyacophila nubila* | 0 | 0 | 0 | 0 | 0 | 0 | 0 | 0 | 1 | 0 | 0 | 0 |
| *Rhyacophila obliterata* | 0 | 0 | 0 | 0 | 0 | 0 | 0 | 0 | 1 | 0 | 0 | 0 |
| *Rhyacophila praemorsa* | 0 | 0 | 0 | 0 | 0 | 0 | 0 | 0 | 1 | 0 | 0 | 0 |
| *Rhyacophila tristis* | 0 | 0 | 0 | 0 | 0 | 0 | 0 | 0 | 1 | 0 | 0 | 0 |
| *Agapetus delicatulus* | 0 | 0 | 0 | 0 | 0 | 0 | 0 | 0 | 0 | 0 | 0 | 0 |
| *Agapetus fuscipes* | 0 | 0 | 0 | 0 | 0 | 0 | 0 | 0 | 0 | 0 | 0 | 0 |
| *Agapetus ochripes* | 0 | 0 | 0 | 0 | 0 | 0 | 0 | 0 | 0 | 0 | 0 | 0 |
| *Glossosoma boltoni* | 0 | 0 | 0 | 0 | 0 | 0 | 0 | 0 | 0 | 0 | 0 | 0 |
| *Allotrichia pallicornis* | 1 | 0 | 0 | 0 | 0 | 0 | 0 | 0 | 0 | 0 | 0 | 0 |
| *Burminoptila bemeneha* | 0 | 0 | 0 | 0 | 0 | 0 | 0 | 0 | 0 | 0 | 0 | 0 |
| *Copulariella ramus* | 0 | 0 | 0 | 0 | 0 | 0 | 0 | 0 | 0 | 0 | 0 | 0 |
| *Hydroptila forcipata* | 1 | 0 | 0 | 0 | 0 | 0 | 0 | 0 | 0 | 0 | 0 | 0 |
| *Hydroptila sparsa* | 1 | 0 | 0 | 0 | 0 | 0 | 0 | 0 | 0 | 0 | 0 | 0 |
| *Ithytrichia lamellaris* | 1 | 0 | 0 | 0 | 0 | 0 | 0 | 0 | 0 | 0 | 0 | 0 |
| *Adicella reducta* | 1 | 0 | 1 | 0 | 0 | 0 | 0 | 0 | 0 | 1 | 1 | 0 |
| *Athripsodes albifrons* | 1 | 0 | 1 | 0 | 0 | 0 | 0 | 0 | 0 | 1 | 1 | 0 |
| *Athripsodes bilineatus* | 1 | 0 | 1 | 0 | 0 | 0 | 0 | 0 | 0 | 1 | 1 | 0 |
| *Athripsodes cinereus* | 1 | 0 | 1 | 0 | 0 | 0 | 0 | 0 | 0 | 1 | 1 | 0 |
| *Ceraclea annulicornis* | 1 | 0 | 1 | 0 | 0 | 0 | 0 | 0 | 0 | 1 | 1 | 0 |
| *Ceraclea dissimilis* | 1 | 0 | 1 | 0 | 0 | 0 | 0 | 0 | 0 | 1 | 1 | 0 |
| *Mystacides azurea* | 1 | 0 | 1 | 0 | 0 | 0 | 0 | 0 | 0 | 1 | 1 | 0 |
| *Mystacides longicornis* | 1 | 0 | 1 | 0 | 0 | 0 | 0 | 0 | 0 | 1 | 1 | 0 |
| *Mystacides nigra* | 1 | 0 | 1 | 0 | 0 | 0 | 0 | 0 | 0 | 1 | 1 | 0 |
| *Oecetis lacustris* | 1 | 0 | 1 | 0 | 0 | 0 | 0 | 0 | 0 | 1 | 1 | 0 |
| *Oecetis ochracea* | 1 | 0 | 1 | 0 | 0 | 0 | 0 | 0 | 0 | 1 | 1 | 0 |
| *Molanna angustata* | 1 | 0 | 0 | 0 | 0 | 0 | 2 | 0 | 0 | 1 | 1 | 0 |
| *Odontocerum albicorne* | 1 | 0 | 0 | 0 | 0 | 0 | 0 | 0 | 0 | 1 | 1 | 0 |
| *Oecismus monedula* | 0 | 1 | ? | 0 | 0 | 1 | 0 | 0 | 0 | 1 | 1 | 0 |
| *Sericostoma flavivorne* | 0 | 1 | ? | 0 | 0 | 1 | 0 | 0 | 0 | 1 | 1 | 0 |
| *Sericostoma personatum* | 0 | 1 | ? | 0 | 0 | 1 | 0 | 0 | 0 | 1 | 1 | 0 |
| *Micrasema longulum* | 0 | 1 | ? | 0 | 0 | 1 | 0 | 0 | 0 | 1 | 1 | 0 |
| *Lepidostoma basale* | 1 | 0 | ? | 0 | 0 | 1 | 2 | 0 | 0 | 0 | 1 | 1 |
| *Lepidostoma hirtum* | 1 | 0 | ? | 0 | 0 | 1 | 2 | 0 | 0 | 0 | 1 | 1 |
| *Goera pilosa* | 1 | 0 | ? | 0 | 0 | 1 | 2 | 0 | 0 | 0 | 1 | 1 |
| *Silo nigricornis* | 1 | 0 | ? | 0 | 0 | 1 | 2 | 0 | 0 | 0 | 1 | 1 |
| *Silo pallipes* | 1 | 0 | ? | 0 | 0 | 1 | 2 | 0 | 0 | 0 | 1 | 1 |
| *Silo piceus* | 1 | 0 | ? | 0 | 0 | 1 | 2 | 0 | 0 | 0 | 1 | 1 |
| *Drusus annulatus* | 0 | 0 | ? | 0 | 0 | 1 | 2 | 1 | 0 | 0 | 1 | 1 |
| *Drusus discolor* | 0 | 0 | ? | 0 | 0 | 1 | 2 | 1 | 0 | 0 | 1 | 1 |
| *Ecclisopteryx dalecarlica* | 0 | 0 | ? | 0 | 0 | 1 | 2 | 1 | 0 | 0 | 1 | 1 |
| *Ecclisopteryx guttulata* | 0 | 0 | ? | 0 | 0 | 1 | 2 | 1 | 0 | 0 | 1 | 1 |
| *Anabolia nervosa* | 0 | 0 | ? | 0 | 0 | 1 | 2 | 1 | 0 | 0 | 1 | 1 |
| *Glyphotaelius pellucidus* | 0 | 0 | ? | 0 | 0 | 1 | 2 | 1 | 0 | 0 | 1 | 1 |
| *Limnephilus centralis* | 0 | 0 | ? | 0 | 0 | 1 | 2 | 1 | 0 | 0 | 1 | 1 |
| *Limnephilus flavicornis* | 0 | 0 | ? | 0 | 0 | 1 | 2 | 1 | 0 | 0 | 1 | 1 |
| *Limnephilus lunatus* | 0 | 0 | ? | 0 | 0 | 1 | 2 | 1 | 0 | 0 | 1 | 1 |
| *Allogamus auricollis* | 0 | 0 | ? | 0 | 0 | 1 | 2 | 1 | 0 | 0 | 1 | 1 |
| *Halesus digitatus* | 0 | 0 | ? | 0 | 0 | 1 | 2 | 1 | 0 | 0 | 1 | 1 |
| *Micropterna lateralis* | 0 | 0 | ? | 0 | 0 | 1 | 2 | 1 | 0 | 0 | 1 | 1 |
| *Parachiona picicornis* | 0 | 0 | ? | 0 | 0 | 1 | 2 | 1 | 0 | 0 | 1 | 1 |
| *Potamophylax nigricornis* | 0 | 0 | ? | 0 | 0 | 1 | 2 | 1 | 0 | 0 | 1 | 1 |
| *Stenophylax permistus* | 0 | 0 | ? | 0 | 0 | 1 | 2 | 1 | 0 | 0 | 1 | 1 |
| *Annitella obscurata* | 0 | 0 | ? | 0 | 0 | 1 | 2 | 1 | 0 | 0 | 1 | 1 |
| *Chaetopteryx villosa* | 0 | 0 | ? | 0 | 0 | 1 | 2 | 1 | 0 | 0 | 1 | 1 |

| Number | 13 | 14 | 15 | 16 | 17 | 18 | 19 | 20 | 21 | 22 | 23 | 24 |
| --- | --- | --- | --- | --- | --- | --- | --- | --- | --- | --- | --- | --- |
| *Cheumatopsyche lepida* | 2 | 0 | 0 | 0 | 1 | 0 | 0 | 0 | 0 | 0 | 0 | 0 |
| *Hydropsyche angustipennis* | 2 | 0 | 0 | 0 | 1 | 0 | 0 | 0 | 0 | 0 | 0 | 0 |
| *Hydropsyche pellucidula* | 2 | 0 | 0 | 0 | 1 | 0 | 0 | 0 | 0 | 0 | 0 | 0 |
| *Hydropsyche saxonica* | 2 | 0 | 0 | 0 | 1 | 0 | 0 | 0 | 0 | 0 | 0 | 0 |
| *Hydropsyche siltalai* | 2 | 0 | 0 | 0 | 1 | 0 | 0 | 0 | 0 | 0 | 0 | 0 |
| *Philopotamus ludificatus* | 0 | 0 | 0 | 0 | 0 | 0 | 0 | 0 | 0 | 0 | 0 | 0 |
| *Philopotamus montanus* | 0 | 0 | 0 | 0 | 0 | 0 | 0 | 0 | 0 | 0 | 0 | 0 |
| *Wormaldia occipitalis* | 0 | 0 | 0 | 0 | 0 | 0 | 0 | 0 | 0 | 0 | 0 | 0 |
| *Cymus trimaculatus* | 0 | 0 | 0 | 0 | 0 | 0 | 1 | 0 | 0 | 0 | 0 | 0 |
| *Plectrocnemia conspersa* | 0 | 0 | 0 | 0 | 0 | 0 | 1 | 0 | 0 | 0 | 0 | 0 |
| *Polycentropus flavomaculatus* | 0 | 0 | 0 | 0 | 0 | 0 | 1 | 0 | 0 | 0 | 0 | 0 |
| *Polycentropus irroratus* | 0 | 0 | 0 | 0 | 0 | 0 | 1 | 0 | 0 | 0 | 0 | 0 |
| *Lype phaeopa* | 0 | 0 | 0 | 0 | 0 | 0 | 1 | 1 | 0 | 0 | 1 | 0 |
| *Lype reducta* | 0 | 0 | 0 | 0 | 0 | 0 | 1 | 1 | 0 | 0 | 1 | 0 |
| *Palerasnitsynus queqiaoi* | 0 | 0 | 0 | 0 | 0 | 0 | 1 | 0 | 0 | 0 | 1 | 0 |
| *Palerasnitsynus qixi* | 0 | 0 | 0 | 0 | 0 | 0 | 1 | 0 | 0 | 0 | 1 | 1 |
| *Psychomyia pusilla* | 0 | 0 | 0 | 0 | 0 | 0 | 1 | 1 | 0 | 0 | 1 | 0 |
| *Tinodes waeneri* | 0 | 0 | 0 | 0 | 0 | 0 | 1 | 1 | 0 | 0 | 1 | 0 |
| *Ecnomus tenellus* | 0 | 0 | 0 | 1 | 0 | 0 | 1 | 1 | 0 | 0 | 0 | 0 |
| *Rhyacophila dorsalis* | 2 | 0 | 0 | 0 | 0 | 1 | 2 | 3 | 1 | 0 | 0 | 0 |
| *Rhyacophila fasciata* | 2 | 0 | 0 | 0 | 0 | 1 | 2 | 3 | 1 | 0 | 0 | 0 |
| *Rhyacophila nubila* | 2 | 0 | 0 | 0 | 0 | 1 | 2 | 3 | 1 | 0 | 0 | 0 |
| *Rhyacophila obliterata* | 2 | 0 | 0 | 0 | 0 | 1 | 2 | 3 | 1 | 0 | 0 | 0 |
| *Rhyacophila praemorsa* | 2 | 0 | 0 | 0 | 0 | 1 | 2 | 3 | 1 | 0 | 0 | 0 |
| *Rhyacophila tristis* | 2 | 0 | 0 | 0 | 0 | 1 | 2 | 3 | 1 | 0 | 0 | 0 |
| *Agapetus delicatulus* | 2 | 0 | 0 | 1 | 0 | 1 | 2 | 2 | 1 | 0 | 0 | 0 |
| *Agapetus fuscipes* | 2 | 0 | 0 | 1 | 0 | 1 | 2 | 2 | 1 | 0 | 0 | 0 |
| *Agapetus ochripes* | 2 | 0 | 0 | 1 | 0 | 1 | 2 | 2 | 1 | 0 | 0 | 0 |
| *Glossosoma boltoni* | 2 | 0 | 0 | 1 | 0 | 1 | 2 | 2 | 1 | 0 | 0 | 0 |
| *Allotrichia pallicornis* | ? | 0 | 0 | 0 | 0 | 1 | 0 | 2 | 1 | 1 | 0 | 1 |
| *Burminoptila bemeneha* | ? | 0 | 0 | 0 | 0 | 1 | 0 | 2 | 1 | 0 | 0 | 1 |
| *Copulariella ramus* | ? | 0 | 0 | 0 | 0 | 1 | 2 | 2 | 1 | 0 | 0 | 1 |
| *Hydroptila forcipata* | ? | 0 | 0 | 0 | 0 | 1 | 0 | 2 | 1 | 1 | 0 | 1 |
| *Hydroptila sparsa* | ? | 0 | 0 | 0 | 0 | 1 | 0 | 2 | 1 | 1 | 0 | 1 |
| *Ithytrichia lamellaris* | ? | 0 | 0 | 0 | 0 | 1 | 0 | 2 | 1 | 1 | 0 | 1 |
| *Adicella reducta* | 1 | 0 | 0 | 1 | 2 | 1 | 2 | 4 | 1 | 0 | 0 | 0 |
| *Athripsodes albifrons* | 1 | 0 | 0 | 1 | 2 | 1 | 2 | 4 | 1 | 0 | 0 | 0 |
| *Athripsodes bilineatus* | 1 | 0 | 0 | 1 | 2 | 1 | 2 | 4 | 1 | 0 | 0 | 0 |
| *Athripsodes cinereus* | 1 | 0 | 0 | 1 | 2 | 1 | 2 | 4 | 1 | 0 | 0 | 0 |
| *Ceraclea annulicornis* | 1 | 0 | 0 | 1 | 2 | 1 | 2 | 4 | 1 | 0 | 0 | 0 |
| *Ceraclea dissimilis* | 1 | 0 | 0 | 1 | 2 | 1 | 2 | 4 | 1 | 0 | 0 | 0 |
| *Mystacides azurea* | 1 | 0 | 0 | 1 | 2 | 1 | 2 | 4 | 1 | 0 | 0 | 0 |
| *Mystacides longicornis* | 1 | 0 | 0 | 1 | 2 | 1 | 2 | 4 | 1 | 0 | 0 | 0 |
| *Mystacides nigra* | 1 | 0 | 0 | 1 | 2 | 1 | 2 | 4 | 1 | 0 | 0 | 0 |
| *Oecetis lacustris* | 1 | 0 | 0 | 1 | 2 | 1 | 2 | 4 | 1 | 0 | 0 | 0 |
| *Oecetis ochracea* | 1 | 0 | 0 | 1 | 2 | 1 | 2 | 4 | 1 | 0 | 0 | 0 |
| *Molanna angustata* | 0 | 0 | 0 | 1 | 1 | 1 | 1 | 4 | 1 | 0 | 1 | 0 |
| *Odontocerum albicorne* | 0 | 0 | 0 | 0 | 1 | 1 | 2 | 0 | 1 | 0 | 0 | 1 |
| *Oecismus monedula* | 0 | 0 | 0 | 0 | 1 | 1 | 2 | 3 | 1 | 0 | 0 | 1 |
| *Sericostoma flavivorne* | 0 | 0 | 0 | 0 | 1 | 1 | 2 | 3 | 1 | 0 | 0 | 1 |
| *Sericostoma personatum* | 0 | 0 | 0 | 0 | 1 | 1 | 2 | 3 | 1 | 0 | 0 | 1 |
| *Micrasema longulum* | 0 | 0 | 0 | 0 | 1 | 1 | 2 | 3 | 1 | 0 | 0 | 1 |
| *Lepidostoma basale* | 1 | 0 | 0 | 0 | 0 | 1 | 2 | 0 | 1 | 0 | 0 | 1 |
| *Lepidostoma hirtum* | 1 | 0 | 0 | 0 | 0 | 1 | 2 | 0 | 1 | 0 | 0 | 1 |
| *Goera pilosa* | 1 | 1 | 1 | 0 | 0 | 1 | 2 | 0 | 1 | 0 | 0 | 1 |
| *Silo nigricornis* | 1 | 1 | 1 | 0 | 0 | 1 | 2 | 0 | 1 | 0 | 0 | 1 |
| *Silo pallipes* | 1 | 1 | 1 | 0 | 0 | 1 | 2 | 0 | 1 | 0 | 0 | 1 |
| *Silo piceus* | 1 | 1 | 1 | 0 | 0 | 1 | 2 | 0 | 1 | 0 | 0 | 1 |
| *Drusus annulatus* | 1 | 1 | 1 | 0 | 1 | 1 | 2 | 0 | 1 | 0 | 0 | 1 |
| *Drusus discolor* | 1 | 1 | 1 | 0 | 1 | 1 | 2 | 0 | 1 | 0 | 0 | 1 |
| *Ecclisopteryx dalecarlica* | 1 | 1 | 1 | 0 | 1 | 1 | 2 | 0 | 1 | 0 | 0 | 1 |
| *Ecclisopteryx guttulata* | 1 | 1 | 1 | 0 | 1 | 1 | 2 | 0 | 1 | 0 | 0 | 1 |
| *Anabolia nervosa* | 1 | 1 | 1 | 0 | 1 | 1 | 2 | 0 | 1 | 0 | 0 | 1 |
| *Glyphotaelius pellucidus* | 1 | 1 | 1 | 0 | 1 | 1 | 2 | 0 | 1 | 0 | 0 | 1 |
| *Limnephilus centralis* | 1 | 1 | 1 | 0 | 1 | 1 | 2 | 0 | 1 | 0 | 0 | 1 |
| *Limnephilus flavicornis* | 1 | 1 | 1 | 0 | 1 | 1 | 2 | 0 | 1 | 0 | 0 | 1 |
| *Limnephilus lunatus* | 1 | 1 | 1 | 0 | 1 | 1 | 2 | 0 | 1 | 0 | 0 | 1 |
| *Allogamus auricollis* | 1 | 1 | 1 | 0 | 1 | 1 | 2 | 0 | 1 | 0 | 0 | 1 |
| *Halesus digitatus* | 1 | 1 | 1 | 0 | 1 | 1 | 2 | 0 | 1 | 0 | 0 | 1 |
| *Micropterna lateralis* | 1 | 1 | 1 | 0 | 1 | 1 | 2 | 0 | 1 | 0 | 0 | 1 |
| *Parachiona picicornis* | 1 | 1 | 1 | 0 | 1 | 1 | 2 | 0 | 1 | 0 | 0 | 1 |
| *Potamophylax nigricornis* | 1 | 1 | 1 | 0 | 1 | 1 | 2 | 0 | 1 | 0 | 0 | 1 |
| *Stenophylax permistus* | 1 | 1 | 1 | 0 | 1 | 1 | 2 | 0 | 1 | 0 | 0 | 1 |
| *Annitella obscurata* | 1 | 1 | 1 | 0 | 1 | 1 | 2 | 0 | 1 | 0 | 0 | 1 |
| *Chaetopteryx villosa* | 1 | 1 | 1 | 0 | 1 | 1 | 2 | 0 | 1 | 0 | 0 | 1 |

| Number | 25 | 26 | 27 | 28 | 29 | 30 |
| --- | --- | --- | --- | --- | --- | --- |
| *Cheumatopsyche lepida* | 0 | 1 | 1 | 0 | 1 | 1 |
| *Hydropsyche angustipennis* | 0 | 1 | 1 | 0 | 1 | 1 |
| *Hydropsyche pellucidula* | 0 | 1 | 1 | 0 | 1 | 1 |
| *Hydropsyche saxonica* | 0 | 1 | 1 | 0 | 1 | 1 |
| *Hydropsyche siltalai* | 0 | 1 | 1 | 0 | 1 | 1 |
| *Philopotamus ludificatus* | 0 | 1 | 2 | 0 | 1 | 0 |
| *Philopotamus montanus* | 0 | 1 | 0 | 0 | 1 | 0 |
| *Wormaldia occipitalis* | 0 | 1 | 0 | 0 | 1 | 0 |
| *Cymus trimaculatus* | 0 | 0 | 1 | 0 | 1 | 0 |
| *Plectrocnemia conspersa* | 0 | 0 | 1 | 0 | 1 | 0 |
| *Polycentropus flavomaculatus* | 0 | 0 | 1 | 0 | 1 | 0 |
| *Polycentropus irroratus* | 0 | 0 | 1 | 0 | 1 | 0 |
| *Lype phaeopa* | 0 | 1 | 1 | 0 | 1 | 0 |
| *Lype reducta* | 0 | 1 | 1 | 0 | 1 | 0 |
| *Palerasnitsynus queqiaoi* | ? | 1 | ? | 0 | 1 | 0 |
| *Palerasnitsynus qixi* | ? | 1 | ? | 0 | 1 | 0 |
| *Psychomyia pusilla* | 0 | 1 | 1 | 0 | 1 | 0 |
| *Tinodes waeneri* | 0 | 1 | 1 | 0 | 1 | 0 |
| *Ecnomus tenellus* | 0 | 0 | 0 | 0 | 1 | 0 |
| *Rhyacophila dorsalis* | 1 | 0 | 0 | 1 | 0 | 0 |
| *Rhyacophila fasciata* | 1 | 0 | 0 | 1 | 0 | 0 |
| *Rhyacophila nubila* | 1 | 0 | 0 | 1 | 0 | 0 |
| *Rhyacophila obliterata* | 1 | 0 | 0 | 1 | 0 | 0 |
| *Rhyacophila praemorsa* | 1 | 0 | 0 | 1 | 0 | 0 |
| *Rhyacophila tristis* | 1 | 0 | 0 | 1 | 0 | 0 |
| *Agapetus delicatulus* | 1 | 1 | 0 | 1 | 0 | 0 |
| *Agapetus fuscipes* | 1 | 1 | 0 | 1 | 0 | 0 |
| *Agapetus ochripes* | 1 | 1 | 0 | 1 | 0 | 0 |
| *Glossosoma boltoni* | 1 | 1 | 0 | 1 | 0 | 0 |
| *Allotrichia pallicornis* | 1 | 4 | 0 | 1 | 0 | 0 |
| *Burminoptila bemeneha* | ? | 4 | 0 | 1 | 0 | 0 |
| *Copulariella ramus* | ? | 4 | 0 | 1 | 0 | 0 |
| *Hydroptila forcipata* | 1 | 4 | 0 | 1 | 0 | 0 |
| *Hydroptila sparsa* | 1 | 4 | 0 | 1 | 0 | 0 |
| *Ithytrichia lamellaris* | 1 | 4 | 0 | 1 | 0 | 0 |
| *Adicella reducta* | 1 | 3 | 2 | 1 | 0 | 0 |
| *Athripsodes albifrons* | 1 | 3 | 2 | 1 | 0 | 0 |
| *Athripsodes bilineatus* | 1 | 3 | 2 | 1 | 0 | 0 |
| *Athripsodes cinereus* | 1 | 3 | 2 | 1 | 0 | 0 |
| *Ceraclea annulicornis* | 1 | 3 | 2 | 1 | 0 | 0 |
| *Ceraclea dissimilis* | 1 | 3 | 2 | 1 | 0 | 0 |
| *Mystacides azurea* | 1 | 3 | 2 | 1 | 0 | 0 |
| *Mystacides longicornis* | 1 | 3 | 2 | 1 | 0 | 0 |
| *Mystacides nigra* | 1 | 3 | 2 | 1 | 0 | 0 |
| *Oecetis lacustris* | 1 | 3 | 2 | 1 | 0 | 0 |
| *Oecetis ochracea* | 1 | 3 | 2 | 1 | 0 | 0 |
| *Molanna angustata* | 0 | 1 | 2 | 1 | 0 | 0 |
| *Odontocerum albicorne* | 0 | 1 | 1 | 1 | 0 | 0 |
| *Oecismus monedula* | 1 | 2 | 2 | 1 | 0 | 0 |
| *Sericostoma flavivorne* | 1 | 2 | 2 | 1 | 0 | 0 |
| *Sericostoma personatum* | 1 | 2 | 2 | 1 | 0 | 0 |
| *Micrasema longulum* | 1 | 2 | 2 | 1 | 0 | 0 |
| *Lepidostoma basale* | 1 | 1 | 0 | 1 | 0 | 0 |
| *Lepidostoma hirtum* | 1 | 1 | 0 | 1 | 0 | 0 |
| *Goera pilosa* | 1 | 1 | 0 | 1 | 0 | 0 |
| *Silo nigricornis* | 1 | 1 | 0 | 1 | 0 | 0 |
| *Silo pallipes* | 1 | 1 | 0 | 1 | 0 | 0 |
| *Silo piceus* | 1 | 1 | 0 | 1 | 0 | 0 |
| *Drusus annulatus* | 1 | 3 | 0 | 1 | 0 | 0 |
| *Drusus discolor* | 1 | 3 | 0 | 1 | 0 | 0 |
| *Ecclisopteryx dalecarlica* | 1 | 2 | 0 | 1 | 0 | 0 |
| *Ecclisopteryx guttulata* | 1 | 2 | 0 | 1 | 0 | 0 |
| *Anabolia nervosa* | 1 | 3 | 0 | 1 | 0 | 0 |
| *Glyphotaelius pellucidus* | 1 | 2 | 0 | 1 | 0 | 0 |
| *Limnephilus centralis* | 1 | 2 | 0 | 1 | 0 | 0 |
| *Limnephilus flavicornis* | 1 | 2 | 0 | 1 | 0 | 0 |
| *Limnephilus lunatus* | 1 | 2 | 0 | 1 | 0 | 0 |
| *Allogamus auricollis* | 1 | 3 | 0 | 1 | 0 | 0 |
| *Halesus digitatus* | 1 | 3 | 0 | 1 | 0 | 0 |
| *Micropterna lateralis* | 1 | 2 | 0 | 1 | 0 | 0 |
| *Parachiona picicornis* | 1 | 2 | 0 | 1 | 0 | 0 |
| *Potamophylax nigricornis* | 1 | 2 | 0 | 1 | 0 | 0 |
| *Stenophylax permistus* | 1 | 2 | 0 | 1 | 0 | 0 |
| *Annitella obscurata* | 1 | 3 | 0 | 1 | 0 | 0 |
| *Chaetopteryx villosa* | 1 | 3 | 0 | 1 | 0 | 0 |

**SI References**

Botosaneanu L. On a false and a genuine caddisfly from Burmese amber (Insecta: Trichoptera, Homoptera). *Bulletin Zoölogisch Museum, Universiteit van Amsterdam* 1981; **8**: 73–78.

Botosaneanu L, Johnson RO and Dillon PR. New caddisflies (Insecta: Trichoptera) from Upper Cretaceous amber of New Jersey, USA. *Polskie Pismo Entomologiczne* 1998; **67**: 219–231.

Cockerell TDA. Eocene Insects from the Rocky Mountains. *Proceedings of the United States National Museum* 1921; **57**: 233–260.

Erickson JM. *Trichopterodomus leonardi*, a new genus and species of Psychomyiid caddisfly (Insecta: Trichoptera) represented by retreats from the Paleocene of North Dakota. *J Paleontol* 1983; **57**: 560–567.

Hagen H. Die im Bernstein befindlichen Neuropteren der Vorwelt bearbeitet von F.J. Pictet-Baraban und Dr. H. Hagen, In Berendt, G.C. (ed.). *Die im Bernstein Befindlichen Organischen Reste der Vorwelt Gesammelt in Verbindung mit Mehreren Bearbeitet und Herausgegeben*, Bd. 2. Berlin. **2**: 41–125.

Melnitsky SI and Ivanov VD. New species of caddisflies (Insecta: Trichoptera) from the Rovno amber, Eocene of Ukraine. *Paleontol J* 2010; **44**: 303–311.

Melnitsky SI and Ivanov VD. Seven new species of caddisflies (Insecta: Trichoptera) from the Rovno amber (Eocene of Ukraine). *Paleontol J* 2013; **47**: 283–291.

Melnitsky SI and Ivanov VD. New species of caddisflies (Insecta, Trichoptera) from the Rovno Amber. *Zoosymposia* 2016; **10**: 278–291.

Mey W. Die Köcherfliegen des Sächsischen Bernsteins (III) (Trichoptera). *Deutsche Entomologische Zeitschrift* 1988; **35**: 299–309.

Mey W. Die Köcherfliegen des Sächsischen Bernsteins (I) (Trichoptera). *Deutsche Entomologische Zeitschrift* 1985; **32**: 275–278.

Pictet FJ and Hagen H. Die im Bernstein befindlichen Neuropteren der Vorwelt. *Die in Bernstein Befindlichen Organischen Reste der Vorwelt Gesammelt in Verbindung mit Mehreren Bearbeitetet und Herausgegeben* 1856; **2**: 41–126.

Ulmer G. Die Trichopteren des baltischen Bernsteins. *Beiträge zur Naturkunde Preussens* 1912; **10**: 1–380.

Wells A and Wichard W. Caddisflies of Dominican amber VI. Hydroptilidae (Trichoptera). *Stud Neotrop Fauna E*. 1989; **24**: 41–51.

Wichard W. Overview of the caddisfies (Insecta, Trichoptera) in mid-Cretaceous Burmese amber. *Cretaceous Res* 2021; **119**: 1–15.

Wichard W. Köcherfliegen des Dominikanischen Bernsteins I. *Ochrotrichia doehleri* sp. nov. (Trichoptera, Hydroptilidae). Mitteilungen der Münchner Entomologischen Gesellschaft 1981; **71**: 161–162.

Wichard W. Overview and Descriptions of Trichoptera in Baltic Amber: Spicipalpia and Integripalpia. Remagen: Kessel, 2013.

Wichard W, P. Müller and B. Wang, The psychomyiid genus *Palerasnitsynus* (Insecta, Trichoptera) in mid-Cretaceous Burmese amber. *Palaeodiversity* 2018; 11: 151–166.

Wichard W, Ross E and Ross AJ. *Palerasnitsynus* gen. n. (Trichoptera, Psychomyiidae) from Burmese amber. *ZooKeys* 2011; **130**: 323–330.
